# Supplementary material for: Does Protein Glycation Impact on the Drought-Related Changes in Metabolism and Nutritional Properties of Mature Pea (Pisum sativum L.) Seeds?
Source: Int J Mol Sci. 2020 Jan 15;21(2):567. doi: 10.3390/ijms21020567 (PMC7013545; doi:10.3390/ijms21020567)
Supplement: Supplementary file 1 [file ijms-21-00567-s001.zip › ijms-678179-SI/200101_Leonova_et_al_Supplementary_1.pdf]

Drought-related changes in the metabolism and nutritional properties of mature pea (*Pisum sativum* L.) seeds in the context of protein glycation

Tatiana Leonova,[1,2] Veronika Popova,[1,3] Alexander Tsarev,[1,2] Christian Henning,[4] Kristina Antonova,[1,2] Nadezhda Rogovskaya,[5] Maria Vikhnina,[1,2] Tim Baldensperger,[4] Alena Soboleva,[1,2] Ekaterina Dinastia,[1,2,6] Mandy Dorn,[2] Olga Shiroglasova,[3] Tatiana Grishina,[1] Gerd U. Balcke,[7] Christian Ihling,[8] Galina Smolikova,[3] Sergei Medvedev,[3] Vladimir A. Zhukov,[9] Vladimir Babakov,[5] Igor A. Tikhonovich,[9,10] Marcus A. Glomb,[4] Tatiana Bilova[2,3] and Andrej Frolov[1,2*]

Supplementary information 1

[1]Department of Biochemistry, St. Petersburg State University, [2]Department of Bioorganic Chemistry, Leibniz Institute of Plant Biochemistry, [3]Department of Plant Physiology and Biochemistry, St. Petersburg State University, [4]Institute of Chemistry - Food Chemistry, Martin-Luther Universität Halle-Wittenberg, [5]Research Institute of Hygiene, Occupational Pathology and Human Ecology, [6]Postovsky Institute of Organic Synthesis of Ural Division of Russian Academy of Sciences, [7]Department of Metabolic and Cell Biology, Leibniz Institute of Plant Biochemistry, [8]Department of Pharmaceutical Chemistry and Bioanalytics, Institute of Pharmacy, Martin-Luther Universität Halle-Wittenberg, [9]All-Russia Research Institute for Agricultural Microbiology and [10]Department of Genetics and Biotechnology, St. Petersburg State University

*Corresponding author:
Dr. Andrej Frolov,
Leibniz Institute of Plant Biochemistry
Department of Bioorganic Chemistry
Weinberg 3
06120, Halle (Saale), Germany
Tel. +49 (0) 345 5582 1350
Fax. +49 (0) 345 5582 1359
Email: afrolov@ipb-halle.de

Directory

Protocols.....S1-6

Protocol S1-1 Isolation of carboxypeptidase Y from yeast *Saccharomyces cerevisiae*.....S1-6

Protocol S1-2 Identification of carboxypeptidase Y from yeast *Saccharomyces cerevisiae*... S1-8

Protocol S1-3 Determination of hydrogen peroxide contents.....S1-9

Protocol S1-4 Determination of lipid hydroperoxide contents.....S1-10

Protocol S1-5 Determination of malondialdehyde (MDA) contents.....S1-11

Protocol S1-6 Determination of ascorbic and dehydroascorbic acid contents.....S1-12

Protocol S1-7 Separation of proteins by SDS-PAGE.....S1-13

Protocol S1-8 Removal of SDS from protein hydrolyzates by solid phase extraction (SPE).S1-14

Tables.....S1-15

Table S1-1 Drought-regulated (at least 1.5-fold) thermally stabile primary metabolites of *Pisum sativum* L. seeds analyzed by untargeted gas chromatography-electron ionization-quadrupole mass spectrometry (GC-EI-Q-MS) approach.....S1-15

Table S1-2 Drought-regulated thermally stabile primary metabolites of Pisum sativum L. seeds.....S1-17

Table S1-3 Protein recoveries and total UV densities for individual pea protein samples separated by SDS-PAGE.....S1-26

Table S1-4 Proteins identified in the tryptic digests obtained from isolated and reference carboxypeptidase Y of yeast *Saccharomyces cerevisiae*.....S1-27

Table S1-5 The conditions of ultrahigh performance liquid chromatographic (UHPLC) separation and the settings for electrospray ionization-triple quadrupole-tandem mass

spectrometry (ESI-QqQ-MS/MS) used for analysis of abscisic acid (ABA) in methanolic extracts of pea (*Pisum sativum* L., cultivar SGE) seeds....................S1-34

Table S1-6 Gas chromatographic (GC) separation conditions and electron ionization-quadrupole-mass spectrometry (EI-Q-MS) settings for analysis of *Pisum sativum* L. primary thermally stabile metabolites....................S1-37

Table S1-7 The conditions of ion pair-reversed phase ultrahigh performance liquid chromatographic (IP-RP-UHPLC) separation and the settings for electrospray ionization-triple quadrupole-tandem mass spectrometry (ESI-QqQ-MS/MS) used for analysis of *Pisum sativum* L. anionic primary thermo labile metabolites....................S1-38

Table S1-8 The conditions of ultrahigh performance liquid chromatographic (UHPLC) separation and the settings for electrospray ionization-quadrupole-time of flight mass spectrometry (ESI-QqTOF-MS) applied for the analysis of *Pisum sativum* L. semi-polar secondary metabolites....................S1-46

Table S1-9 Composition of the alkane mixture used for determination of Kovats retention time indices (RIs) S1-49

Figures....................S1-51

Figure S1-1 Cultivation of pea (*Pisum sativum* L., cultivar SGE) plants....................S1-51

Figure S1-2 Optimization of experimental drought conditions applied to pea plants pea (*Pisum sativum* L., cultivar SGE) plants at the stage of seed maturation....................S1-52

Figure S1-3 Morphology and anatomy of pea (*Pisum sativum* L., cultivar SGE) seed..........S1-53

Figure S1-4 Characterization of the stress response in pea (*Pisum sativum* L., cultivar SGE) leaves....................S1-54

Figure S1-5 The results of the principal component analysis (PCA), done for the abundances of the primary thermally stabile polar metabolites..S1-55

Figure S1-6 The results of the principal component analysis (PCA), done for the abundances of the primary thermally labile anionic polar metabolites..S1-56

Figure S1-7 The results of the principal component analysis (PCA), done for the abundances of the anionic semi-polar secondary metabolites...S1-57

Figure S1-8 The results of the principal component analysis (PCA), done for the abundances of the cationic semi-polar secondary metabolites..S1-58

Figure S1-9 Pathway analyses accomplished separately for the drought-regulated primary polar metabolites of pea (*Pisum sativum* L., cultivar SGE) seeds annotated by untargeted GC-EI-Q-MS and targeted IP-RP-UHPLC-MS/MS...S1-59

Figure S1-10 The PLS-DA model and loadings plot, designed for 1092 of 2667 semi-polar features annotated by RP-UHPLC-QqTOF-MS operated in positive SWATH mode............S1-61

Figure S1-11 Hierarchical clustering analysis of 51 up-regulated and 15 down-regulated drought-related semi-polar metabolite MS features using the corresponding MS/MS spectra obtained from RP-UHPLC-QqTOF-MS operated in positive SWATH mode.......................S1-62

Figure S1-12 The PLS-DA model and loadings plot, designed for 207 of 376 semi-polar metabolites identified by RP-UHPLC-QqTOF-MS operated in negative SWATH mode......S1-63

Figure S1-13 Hierarchical cluster analysis (HCA) of 12 up-regulated drought-related semi-polar metabolites (MS^1) using the corresponding MS/MS spectra obtained from RP-UHPLC-QqTOF-MS operated in negative SWATH mode)..S1-64

Figure S1-14 SDS-PAGE electropherograms of pea seed protein before and after exhaustive enzymatic hydrolysis (n=5) S1-65

Figure S1-15 The effects of pea seed protein hydrolyzates on the NF-κB-mediated signaling pathway in SH-SY5Y human neuroblastoma cells: optimization of the applied protein amounts..S1-66

Figure S1-16 The effects of pea seed protein hydrolyzates on the NF-κB-mediated signaling pathway in SH-SY5Y human neuroblastoma cells: optimization of the applied protein amounts..S1-68

Figure S1-17 The effects of pea seed protein hydrolyzates on the NF-κB-mediated signaling pathway in SH-SY5Y human neuroblastoma cells: optimization of incubation times............S1-70

Figure S1-18 SDS-PAGE electropherograms of yeast carboxypeptidase Y purchased from Merck (Sigma-Aldrich GmbH), and carboxypeptidase Y isolated from the yeast *Saccharomyces cerevisiae* according to the protocol of Johansen *et al*...S1-72

References..S1-73

Protocols

Protocol S1-1 Isolation of carboxypeptidase Y from yeast *Saccharomyces cerevisiae* [1]

Carboxypeptidase Y was isolated from baker yeast (*S. cerevisiae*) by affinity chromatography on a Sepharose 4B-Gly-Tyr-azobenzylbutanedioic acid sorbent. The affinity resin was prepared as follows: 25 mg of the sorbent Sepharose 4B were washed, re-suspended in 25 mL water and activated by the addition of 4.25 mg cyanogen bromide under continuous stirring. The pH was maintained at 10.5 by continuous addition of 6 mol/L NaOH, and temperature maintained at 20°C by addition of ice for 10-15 min. Afterwards, the activated Sepharose was filtered, washed with 250 mL water and 125 mL ice-cold 0.1 mol/L $NaHCO_3$ (pH 9.5) and suspended in 25 mL of the same buffer containing 250 mg Gly-Tyr (within 5 min time limit). The resulting solution was incubated at 4°C for 3 days. Afterwards, 50 mg *p*-aminobenzylsuccinic acid was dissolved in 10 mL 1 mol/L HCl, mixed with 5 mL 0.3 mol/L $NaNO_2$, the mixture was stirred for 10 min at 4°C and added to the ice-cold suspension of 25 mL Sepharose-Gly-Tyr. The pH value was adjusted to 9.5. After 3 h of reaction at 4°C the Sepharose derivate turned orange and was washed with 50 mL ice-cold 0.1 mol/L $NaHCO_3$ (pH 9.5) and 250 mL water. The resulted affinity resin could be stored over long periods at 4°C as an aqueous suspension.

Approximately 500 g of compressed *S. cerevisiae* yeast were frozen in liquid nitrogen and ground in a Mixer Mill MM 400 ball mill with a 20 mm stainless steel ball (Retsch, Haan, Germany) at a vibration frequency of 30 Hz for 1 min. The resulted powder was supplemented with 500 mL ether and, one hour later, 750 mL of water was added, before the pH of the solution was adjusted to 7.4 with 1 mol/L NaOH. The resulting suspension was incubated for 24 hours at 25°C and centrifuged (10000 g, 10 min, 4°C) to remove the cell debris. The supernatant was collected, the pH value was adjusted to 5.0, and the solution was incubated for 24 hours at 30°C under constant stirring and centrifuged afterwards (10000 g, 10 min, 4°C). The resulted supernatant was concentrated to 100 mL in a vacuum evaporator (Rotavapor R-114, Büchi, Essen, Germany), and pH was re-adjusted to 5.0. Then, the affinity resin and 100 mL 10 mmol/L

2-(*N*-morpholino)ethansulfonic acid (MES) buffer (pH 5.0) were added to the solution and stirred at 4°C overnight. The resulted suspension was washed with 1 mol/L NaCl containing 10 mmol/L sodium acetate (pH 4.3) until absorbance of the eluate at 280 nm decreased below 0.005 units. The retained carboxypeptidase Y was eluted with 0.01 mol/L sodium phosphate buffer (pH 7.0). The resulted eluate was concentrated and desalted by ultrafiltration (Vivaspin Turbo 3 kDa MWCO filters). The aqueous enzyme solution was frozen and stored at -20°C. The preparation was analyzed by SDS-PAGE (see Protocol S1-7 and Figure S1-1) and by liquid chromatography-mass spectrometry (LC-MS, Protocol S1-2). The commercially available carboxypeptidase Y purchased from Sigma-Aldrich GmbH/Merk (No C3888) served as an analytical reference for SDS-PAGE and LC-MS analysis.

Protocol S1-2 Identification of carboxypeptidase Y from yeast *Saccharomyces cerevisiae*

The protein concentration in the carboxypeptidase Y isolate was determined by 2D Quant Kit according to the manufacturer's instructions. Afterwards, in-gel digestion of proteins was performed as described by Majovski et al [2]. Protein samples (10 µg) were separated by SDS-PAGE (Protocol S1-7 and Figure S1-1). Two gel bands on each lane were excised and destained with 30% (v/v) acetonitrile, 100 mmol/L NH_4HCO_3 (pH 8.5). The samples were sequentially incubated with 10 mmol/L DTT, 100 mmol/L NH_4HCO_3 (pH 8.5) for 30 min at 50°C to reduce disulfide bonds, and then with 54 mmol/L iodoacetamide, 100 mmol/L NH_4HCO_3 (pH 8.5) for 15 min at 22°C in dark to provide alkylation. Protein digestion was performed by adding of 70 µL trypsin (3 ng/µL in 100 mmol/L NH_4HCO_3 solution) and overnight incubation at 37°C. Peptides were extracted from the gel with 35% (v/v) acetonitrile, 0.4 % (v/v) trifluoroacetic acid. After this, the digests were pre-cleaned by solid phase extraction (SPE) on Stage-Tips filled with C_{18} matrix (3M™ Empore Extraction Disks, Pittsburgh, USA) as described by Mamontova *et al* [3]. In detail, the Stage-Tips were pre-conditioned with 100 µL of methanol and equilibrated with 200 µL of 0.1% (v/v) trifluoroacetic acid (twice). After load of the peptide solution, the unbound components were washed out by two portions 200 µL of 0.1% (v/v) aqueous formic acid. Afterwards, retained tryptic peptides were sequentially eluted with 50 µL of 40%, 60%, 80% (v/v) aq. acetonitrile. The combined eluates were dried and reconstituted in aq. 0.1% (v/v) formic acid solution containing decreasing amounts of acetonitrile to obtain its final concentration of 3% (v/v). The mass spectrometric analysis relied on nano-scaled high-performance liquid chromatography coupled on-line to electrospray ionization-linear ion trap-orbital trap hybrid mass spectrometer (nanoHPLC-ESI-LIT-Orbitrap-MS) as described by Mamontova *et al* [4].

Protocol S1-3 Determination of hydrogen peroxide contents

Approximately 100 mg of the plant material were extracted with 1 mL of ice-cold 0.4 mol/L perchloric acid. Samples were vortexed for 30 s and centrifuged (10 000 g, 10 min, 4 °C). The supernatant was neutralized with KOH, diluted four-fold with sodium phosphate buffer (0.1 mol, pH 5.6) and supplemented with ascorbate oxidase (8 units, 2 µL in 4 mmol/L sodium phosphate buffer pH 5.6, 10 min, RT). Afterwards, two aliquots (500 µL each) were transferred to new polypropylene tubes, with one of them treated with catalase (50 units in 2 µL in 4 mmol/L sodium phosphate buffer pH 5.6, 2 min, RT). Both aliquots were supplemented with an equal volume of the FOX reagent (0.2 mmol/L xylenol orange, 200 mmol/L sorbitol, 50 mmol/L H_2SO_4, and 0.5 mmol/L $(NH_4)_2$ $Fe(SO_4)_2$), and incubated for 30 min in the dark before measurement of the Fe(II)-xylenol orange complex absorption at 575 nm. The values obtained for the catalase-treated samples were subtracted from those of the catalase-free ones, to obtain the corrected optical densities. The calibration was performed externally by an H_2O_2 serial dilution series (1–10 µmol/L).

Protocol S1-4 Determination of lipid hydroperoxide contents

Approximately 10 mg of frozen milled plant material were left for 5 min on ice, before 750 µL of ice-cold chloroform-methanol mixture (1:2, v/v) and 150 µL of 0.15 mol/L aq. acetic acid were added and the suspension was vortexed for 30 s. Then, chloroform and water (225 µL each) were added, the suspension was vortexed for 30 s and centrifuged at 3000 g for 5 min. The lower phase was collected, transferred to black polypropylene tubes and dried under nitrogen flow provided by a sample concentrator (Bibby Scientific Limited, Staffordshire, UK) for 30 – 60 min. The residue was reconstituted in 100 µL 0.01% butylated hydroxytoluene (BHT) in methanol and left on ice for 30 min before 900 µL of working FOX reagent (1.0 mmol/L xylenol orange and 2.5 mmol/L ammonium ferrous sulfate in 250 mmol/L H_2SO_4 – 0.01% BHT in methanol, 1 : 9, v/v) was added. After 30 min incubation on ice, absorption was measured at 650 nm against working FOX reagent. Hydroperoxide content was calculated as 13*S*-hydroperoxy-9*Z*, 11*E*-octadecanoic acid equivalents, $\varepsilon = 6.0 \times 10^4\ M^{-1}cm^{-1}$ [5].

Protocol S1-5 Determination of malondialdehyde (MDA) contents

Approximately 25 mg of frozen grinded plant material were left on ice for 3 minutes, before addition of 300 µL 5% (w/v) trichloroacetic acid (TCA), vortexed for 30 s and centrifuged at 10000 g for 20 minutes at 4°C. 250 µL of supernatant were transferred in a new polypropylene tube, and 1000 µL of thiobarbituric acid (TBA) reagent (0.5 % w/v TBA in 20% TCA) were added. The mixture was incubated for 30 min in boiling water bath (95°C). Afterwards, the mixture was cooled on ice to stop the reaction, centrifuged at 1900 g for 10 minutes at 4°C and 1 ml of colored supernatant was used to measure the absorbance at 532 nm against the proper blank (250 µL 5% w/v TCA and 750 µL TBA reagent). The non-specific absorbance at 600 nm was subtracted from the absorbance acquired at 532 nm. The contents of MDA equivalents were calculated with $\varepsilon = 155\ mM^{-1}cm^{-1}$.

Protocol S1-6 Determination of ascorbic and dehydroascorbic acid contents

Approximately 50 mg of frozen plant material were left on ice for 5 min before 0.5 mL of ice-cold 2.5 mol/L $HClO_4$ were added. The suspensions were vortexed for 30 s and centrifuged for 10 min at 10000 g and 4°C. The supernatants were transferred in new polypropylene tubes, neutralized with saturated Na_2CO_3 solution and 10-fold diluted with 0.1 mol/L sodium phosphate buffer (pH 5.6). For determination of ascorbic acid, 500 µL of diluted extract were placed in a quartz cell and absorbance at 265 nm was recoded (Gemini EM microplate reader, Molecular Devices (Germany) GmbH, Biberach, Germany) before 1 u of ascorbate oxidase (i.e. 1 µL in 4 mmol/L sodium phosphate buffer) was added, and absorbance was recoded once more two minutes later. Total ascorbate was quantified after reduction of diluted extract with DTT (3 µL of 3 mol/L solution) for 1 min on ice at the same wavelength. Dehydroascorbic acid was calculated as the difference of the total ascorbate and ascorbic acid contents.

Protocol S1-7 Separation of proteins by SDS-PAGE

Polyacrylamide gel electrophoresis in sodium dodecyl sulfate (SDS-PAGE) was performed with a 12% separating and a 4% stacking gel (T=12%, C=2.65%) [6]. The aliquots of protein (10 μg) or aliquots of enzymatic hydrolysates, corresponding to 30 μg of protein, were dried under reduced pressure and reconstituted in 10 μL of sample buffer, containing 0.02% (w/v) bromophenol blue, 20% (v/v) glycerol, 2% (w/v) SDS, 5% (v/v) β-mercaptoethanol in 50 mmol/L Tris-HCl (pH 6.8) and completely loaded on each lane. After completion of separation (15 min at 90 V, 60 min at 130 V), gels were stained with Coomassie Brilliant Blue G-250 for 1 h. Average densities across individual lanes (expressed in arbitrary units) were determined by ChemiDoc XRS imaging system controlled by Quantity One 1-D analysis software (Bio-Rad Laboratories Ltd). For calculation of relative standard deviations (RSDs), the densities of individual lines were normalized to the gel average value.

Protocol S1-8 Removal of SDS from protein hydrolyzates by solid phase extraction (SPE)

The SPE was accomplished on the reversed phase using Chromabond C_{18} endcapped polypropylene columns and vacuum manifold (operated under the pressure of 850 mbar) to remove the detergent as described by Antonova *et al* [7]. In detail, the cartridges were pre-conditioned with 6 mL of methanol, equilibrated with 6 mL of water, and then the hydrolysates were applied. The fractions, containing amino acids were sequentially eluted with 12 mL of 25 mmol/L aq. ammonium acetate and 12 mL of 100 mmol/L aq. ammonia. The flow-through and both eluate fractions were saved in one 50-mL polypropylene tube and freeze-dried. The residues were sequentially re-constituted in two portions of 0.75 mL of 20% aq. acetonitrile, transferred into 2 mL polypropylene tubes, freeze-dried, and stored afterwards at -20°C.

Tables

Table S1-1 Drought-regulated (at least 1.5-fold) thermally stabile primary metabolites of *Pisum sativum* L. seeds analyzed by untargeted gas chromatography-electron ionization-quadrupole mass spectrometry (GC-EI-Q-MS) approach (t-test *p* value < 0.05, FDR adjusted: p < 0.10).

**Analyte**	**Annotation**	**RI**[a]	**KEGG**[b]	***m/z***[c]	t_R[d]	**FC**[e]	**Raw p**[f]	**FDR**[g]
Homoserine (3TMS)	homoserine[h]	1368	C00263	218	18.61	4.5	0.002	0.019
Unknown 1	-	2966		204	41.09	3.0	0.014	0.055
Unknown 2	-	3971		204	53.34	2.2	0.001	0.019
Unknown 3	oligosaccaride[i]	3200		340	43.58	1.8	0.015	0.056
Unknown 4	carbohydrate [i]	2475		361	35.21	1.8	0.000	0.000
Unknown 5	carbohydrate [i]	2491		361	35.40	1.8	0.000	0.000
Unknown 6	sugar acid [i]	2007		292	28.46	1.7	0.000	0.002
Unknown 7	-	1789		217.05	24.81	1.7	0.022	0.078
Unknown 8	-	1870		174.1	26.2	1.7	0.013	0.056
Unknown 9	-	2987		456	41.33	1.6	0.000	0.008
Unknown 10	-	1841		292	25.71	1.6	0.009	0.040

Unknown 11	-	4068		204.05	54.76	1.6	0.016	0.072
Turanose (1MEOX, 8TMS)	turanose[h]	2837	C19636	243	39.63	1.6	0.000	0.002
Melibiose (1MEOX, 8TMS)	melibiose[h]	2836	C05402	361	39.62	1.5	0.000	0.007
Unknown 12	-	2975		204	41.19	1.5	0.000	0.008
Mannose (1MEOX, 5TMS)	mannose[h]	1926	C00159	319	27.12	0.6	0.024	0.072
Unknown 13	inositol isomer-1 [i]	1988		318	28.15	0.6	0.002	0.019
Unknown 14	inositol isomer-2[i]	1991		318	28.22	0.5	0.002	0.019
Maleic acid (2TMS)	maleic acid[h]	1315	C01384	245	15.37	0.5	0.014	0.055
Unknown 15	-	1886		374	26.47	0.3	0.002	0.019

The analytes were identified by co-elution with authentic standards of by spectral similarity using spectral libraries – NIST 8.0 (National Institute of Standards and Technology) or GMD (GolmMetabolom Database, updated 17/02/2017, http://gmd.mpimp-golm.mpg.de). The seeds were obtained from the plants exposed during two days (at the seed maturation step) to the aqueous medium supplemented with 2.5% (w/v) PEG 8000 (stress, n = 6) in comparison to the seeds of the plants, treated with PEG-free medium (control, n = 6).

[a] RI, Kovats retention Index; [b] the metabolite identical numbers of Kyoto Encyclopedia of Genes and Genomes (KEGG) database; [c] the *m/z* refers to compound-specific fragment ions, selected for quantification by peal areas at characteristic extracted ion chromatograms; [d] retention time (t_R, min); [e] fold change was calculated is abundance (peak areas at characteristic extracted ion chromatograms) ratio drought/control; [f] and [g] *p* values calculated

without (RAW p) and with false discovery rate (FDR) correction by Benjamini-Hochberg method, respectively; [h] and [i] the metabolite annotations were relied on RI, t_R and mass-spectrum of the standard compounds as indicated in Supplementary Information 2, Table S2-1 or relied on *m/z* value(s) characteristic for the mentioned chemical group (some of the characteristic *m/z* values are given as following: *m/z* 361, 319, 217, 103 – carbohydrate and oligosaccharide; *m/z* 103, 292, 333 - sugar acid; *m/z* 305, 318 - inositol isomer), respectively.

Table S1-2 Drought-regulated (at least 1.5-fold, t-test: $p < 0.05$, FDR adjusted: $p < 0.10$) thermally unstable primary metabolites of *Pisum sativum* L. seeds analyzed by targeted ion pair-reversed phase ultrahigh performance liquid chromatography, coupled on-line to electrospray ionization-triple quadrupole-tandem mass spectrometry (IP-RP-UHPLC-ESI-QqQ-MS/MS), performed in negative multiple reaction monitoring (MRM).

**Metabolite**	**Acronim**	**KEGG**[a]	***m/z***[b]	$\mathbf{t_R}$[c]	**FC**[d]	**RAW p**[e]	**FDR**[f]
Dihydroorotic acid	DHO	C00337	157.1	3.57	0.6	0.086	0.096
Adenosine	-	C00212	266.2	2.35	0.5	0.025	0.073
Ribose-1-phosphate	R1P	C00620	229.1	8.40	0.5	0.013	0.071
Phenylalanine	PHE	C00079	164.2	1.66	0.5	0.019	0.071
Sucrose	-	C00089	341.1	0.72	0.5	0.011	0.071
Ascorbic acid	ASC	C00072	175	2.07	0.5	0.018	0.071
Ureidosuccinic acid	CA	C00438	175.1	10.01	0.5	0.083	0.077
Cytidine	-	C00475	242.2	0.81	0.4	0.057	0.086
2-C-Methylerythritol 4-phosphate	MEP	C11434	215	5.61	0.4	0.036	0.071

Lactic acid	LAC	C00186 / C00256	89.1	3.48	0.4	0.026	0.074
Asparagine	ASN	C00152	131.1	0.69	0.4	0.015	0.071
Glycine	GLY	C00037	74.1	0.67	0.4	0.023	0.072
Threonine	THR	C00188	118.1	0.68	0.4	0.062	0.091
2C-Methyl-D-Erythritol 2,4-Cyclodiphosphate	MEcPP	C11453	277	10.85	0.3	0.035	0.075
Serine	SER	C00065	104.1	0.66	0.3	0.010	0.071
Guanosine	-	C00387	282.2	1.47	0.3	0.055	0.085
Glucose	GLC	C00031	178.9	0.70	0.3	0.006	0.071
Glutamine	GLN	C00064	145.1	0.68	0.3	0.038	0.075
Allantoin	ALT	C01551	156.96	0.58	0.3	0.013	0.071
α-Ketoglutaric acid	AKG	C00026	145.01	10.71	0.3	0.032	0.074
Uridine	-	C00299	243.2	1.00	0.3	0.008	0.071
Orotic acid	ORO	C00295	155.1	4.68	0.3	0.050	0.077

Tryptophan	TRP	C00078	203.2	3.37	0.3	0.008	0.071
3-Ureidopropionic acid	CarbamoylAla	C02642	130.9	2.76	0.3	0.033	0.084
Arginine	ARG	C00062	173.2	0.61	0.3	0.010	0.071
Histidine	HIS	C00135	154.2	0.62	0.3	0.009	0.071
Shikimic acid	SHIC	C00493	172.892	2.12	0.3	0.010	0.071
Fumaric acid	FUM	C00122	115.101	10.11	0.3	0.069	0.097
Glucose-1-phosphate	G1P	C00103	259.1	7.13	0.2	0.050	0.077
Lysine	LYS	C00047	145.2	0.69	0.2	0.078	0.097
Tyrosine	TYR	C00082	180.2	0.94	0.2	0.006	0.071
Phytic acid	IP6	C01204	658.555	19.34	0.2	0.031	0.074
Ornithine	ORN	C00077	131.2	1.14	0.2	0.039	0.074
4-Diphosphocytidyl-2-C-methyl-D-erythritol 2-phosphate	CDP-ME-2P	C11436	600.0	15.85	0.2	0.056	0.085

Cyclic guanosine monophosphate	cGMP	C00942	344.2	6.19	0.2	0.042	0.079
Riboflavin-5'-phosphate	FMN	C00061	498.814	0.56	0.2	0.058	0.086
Lactic acid	LAC.1	C00186 / C00256	89.1	3.48	0.2	0.026	0.077
Ribose-5-phosphate	R5P	C00117	229.1	6.20	0.2	0.036	0.077
Phosphocholine	PCHOL	C00588	242.2	0.89	0.2	0.010	0.071
Glutamic acid	GLA	C00025	146	1.26	0.2	0.013	0.071
Sedoheptulose-7-phosphate	S7P	C00281	289.2	6.48	0.2	0.038	0.077
Erythrose-4-phosphate	E4P	C00279	199.1	6.30	0.2	0.030	0.074
Isocitric acid	ISOCIT	C00311	191.1	14.34	0.1	0.015	0.071
1-Diphosinositol pentakisphosphate	IP5	C11174	578.755	19.28	0.1	0.032	0.074
Aspartic acid	ASP	C00049	132	1.43	0.1	0.012	0.071
Xanthosine-5'-phosphate	XAN5P	C00655	363.2	13.14	0.1	0.023	0.072
Citric acid	CIT	C00158	191	14.03	0.1	0.027	0.074

Aconitic acid	ACT	C02341	172.866	13.41	0.1	0.014	0.071
D-Malate	MAL	C00497	133.1	10.10	0.1	0.069	0.074
Guanosine-5'-diphosphate	GDP	C00035	442.2	13.51	0.1	0.012	0.071
Uridine-5'-diphosphate-glucose	UDP-glc	C00029	565.3	10.03	0.1	0.021	0.071
Guanosine-5'-triphosphate	GTP	C00044	522.2	16.12	0.1	0.013	0.071
Adenylosuccinic acid	Adenylo-Suc	C03794	462.3	16.15	0.1	0.023	0.072
Coenzyme A	CoA	C00010	766.1	16.54	0.1	0.089	0.074
Adenosine monophosphate	AMP	C00020	346.2	9.05	0.1	0.019	0.071
S-adenosyl-L-homocysteine	SAH	C00021	383.4	1.46	0.1	0.032	0.074
Flavin adenine dinucleotide	FAD	C00016	784.5	13.12	0.1	0.021	0.071
Adenosine diphosphate glucose	ADP-Glc	C00498	587.864	10.58	0.1	0.034	0.074
Thymidine-5'-diphosphate	dTDP	C00363	401.2	13.45	0.1	0.052	0.084
Adenosine diphosphate ribose	ADP-ribose	C00301	557.94	10.18	0.1	0.013	0.071

Glucosamine-6-phosphate	GA6P	C00352	258.2	1.95	0.1	0.014	0.071
Nicotinic acid ribonucleotide	NAAM	C01185	333.885	19.40	0.1	0.730	0.071
Glutathione	GSH	C00051	306.3	4.31	0.1	0.019	0.071
Deoxythymidine-5'-triphosphate	dTTP	C00459	481.2	16.06	0.1	0.028	0.074
3-Phosphoglyceric acid	3PG	C00597	185.1	13.12	0.1	0.054	0.085
Quinic acid	QUIN	C06746	190.932	3.29	0.1	0.038	0.075
Cytidine monophosphate	CMP	C00055	322.2	7.22	0.1	0.030	0.074
1,4-Dihydronicotinamide adenine dinucleotide	NADH	C00004	664.4	11.26	0.1	0.019	0.071
Inositol-1,3,4,5-tetraphosphate	IP4	C01272	498.704	19.05	0.1	0.024	0.072
S-Acetylcoenzyme A	AcCoA	C00024	807.9	17.60	0.1	0.037	0.075
6-Phosphogluconic acid	6PG	C00345	275	13.93	0.1	0.040	0.077
Glycerophosphoric acid	GLC3P	C00093	171.1	6.61	0.1	0.016	0.071
Glucosamine-1-phosphate	GA1P	C00137	258.2	1.96	0.1	0.008	0.071

5-Phosphoribosyl diphosphate	PRPP	C00119	389.1	17.46	0.1	0.030	0.074
Fructose-6-Phosphate	F6P	C00085	259.1	5.99	0.1	0.042	0.077
Adenosine-5'-diphosphate	ADP	C00008	426.2	13.51	0.1	0.024	0.072
Hydroxymethylglutaroyl-coenzyme A	HMG-CoA	C00356	910.15	18.36	0.1	0.014	0.071
Inositol-1,4,5-triphosphate	IP3	C01245	418.737	17.74	0.1	0.055	0.085
Oxiglutatione	GSSG	C00127	611.6	9.09	0.05	0.013	0.071
Adenosine triphosphate	ATP	C00002	506.2	16.04	0.05	0.018	0.071
Uridine monophosphate	UMP	C00105	323.2	8.01	0.05	0.045	0.077
Glucose-6-phosphate	G6P	C00668	259.1	5.98	0.05	0.043	0.077
NADPH	NADPH	C00005	744.4	16.49	0.05	0.019	0.071
Cytidine-5'-diphosphate	CDP	C00112	402.2	13.15	0.05	0.054	0.085
Cytidine-5'-triphosphate	CTP	C00063	482.2	15.80	0.05	0.027	0.074
Nicotinamide adenine dinucleotide	NAD	C00003	662.4	5.07	0.05	0.034	0.074

Uridine-5'-triphosphate	UTP	C00075	483.1	15.99	0.04	0.031	0.074
Uridine-5'-diphosphate	UDP	C00015	403.2	13.36	0.04	0.046	0.077

The analytes were identified by co-elution with authentic standards. The seeds were obtained from the plants, exposed during two days (at the seed maturation step) to the aqueous medium supplemented with 2.5% (w/v) PEG 8000 (stress, n = 5) in comparison to the seeds of the plants, treated with PEG-free medium (control, n = 5).

[a] the metabolite identical numbers of Kyoto Encyclopedia of Genes and Genomes (KEGG) database; [b] the *m/z* refers to compound-specific fragment ions, selected for quantification by peal areas at characteristic extracted ion chromatograms; [c] retention time (t_R, min); [d] fold change was calculated is abundance (peak areas at characteristic extracted ion chromatograms) ratio drought/control; t_R, retention time [e] and [f] *p* values calculated without (RAW p) and with false discovery rate (FDR) correction by Benjamini-Hochberg method, respectively

Table S1-3 Protein recoveries and total UV densities for individual pea protein samples separated by SDS-PAGE

Sample	Sample weight (g)	Protein concentration (mg/mL)	Protein recovery (mg/g fresh weight)	UV densities (AU)
Control-1	0.419	31.4	89.9	34217
Control-2	0.398	29.9	82.5	35147
Control-3	0.407	26.6	78.4	35596
Control-4	0.420	36.3	103.7	36079
Control-5	0.401	27.4	95.6	36799
Stress-1	0.404	34.3	106.1	37281
Stress-2	0.406	44.8	110.3	37598
Stress-3	0.403	30.9	103.4	39156
Stress-4	0.403	44.4	104.6	39343
Stress-5	0.395	39.3	104.4	39665

Control and Stress denote the seeds of the control and subjected to two-day long drought pea plants (*Pisum sativum* L., cultivar SGE), respectively; AU, arbitrary units.

Table S1-4 Proteins identified in the tryptic digests obtained from isolated and reference carboxypeptidase Y of yeast *Saccharomyces cerevisiae.*

Sample	Accession number	Protein name	Coverage [%]	Number of Peptides	Number of Peptide-Spectrum Match	Number of Unique Peptides	Number of AAs	Score Sequest HT	Number of Peptides (by Search Engine)
Sigma-Aldrich C388 (upper band)	P00729	Carboxypeptidase Y	10,34	9	14	9	532	22,37	9
	Q12306	Ubiquitin-like protein SMT3	46,53	4	5	4	101	18,83	4
	Q02724	Ubiquitin-like-specific protease 1	9,50	5	6	5	621	11,65	5
	P38805	Ribosome production factor 1	5,42	2	2	2	295	3,38	2
	Q02908	Elongator complex protein 3	5,57	1	1	1	557	2,43	1

	Q03281	Inner nuclear membrane protein HEH2	3,92	1	2	1	663	2,19	1
	P32849	DNA repair protein RAD5	0,77	1	1	1	1169	2,19	1
	P25335	Allantoicase	3,79	1	1	1	343	2,12	1
	Q12180	Halotolerance protein 9	1,94	1	1	1	1030	2,06	1
	P43549	Uncharacterized membrane protein YFL054C	2,17	1	1	1	646	2,05	1
Sigma-Aldrich C388 (lower	P00729	Carboxypeptidase Y	13,72	14	74	14	532	122,98	14

band)								
	Q12306	Ubiquitin-like protein SMT3	39,60	3	5	3	101	11,56	3
	P40395	Guanine nucleotide exchange factor subunit RIC1	2,46	2	4	2	1056	5,41	2
	P41896	Transcription initiation factor IIF subunit beta	4,00	1	5	1	400	4,33	1
	P07806	Valine--tRNA ligase, mitochondrial	1,36	1	2	1	1104	3,93	1
	P21372	Pre-mRNA-processing ATP-dependent RNA helicase PRP5	3,89	2	2	2	849	3,66	2
	Q99383	Nuclear polyadenylated RNA-binding protein 4	2,06	1	2	1	534	3,61	1

	P47047	ATP-dependent RNA helicase DOB1	1,86	1	2	1	1073	3,58	1
	P39735	Single-strand annealing weakened protein 1	3,07	1	2	1	261	2,32	1
	Q01846	Structural protein MDM1	2,48	3	3	3	1127	2,22	3
Isolated proteinase (upper band)	P00729	Carboxypeptidase Y	10,90	11	40	11	532	50,18	11
	Q12306	Ubiquitin-like protein SMT3	39,60	3	6	3	101	15,13	3
	Q02724	Ubiquitin-like-specific protease 1	3,06	2	2	2	621	3,08	2

P19358	S-adenosylmethionine synthase 2	5,47	2	2	2	384	3,02	2
P87275	Altered inheritance of mitochondria protein 11	6,57	1	2	1	137	2,43	1
P38283	Inner centromere protein-related protein SLI15	3,44	2	3	2	698	2,37	2
P22137	Clathrin heavy chain	0,97	1	1	1	1653	2,25	1
P47037	Structural maintenance of chromosomes protein 3	0,89	1	1	1	1230	2,14	1
P21264	Phosphoribosylaminoimidazole carboxylase	1,40	1	1	1	571	2,13	1

	P08964	Myosin-1	2,07	3	3	3	1928	2,13	3
Isolated proteinase (lower band)	P07267	Saccharopepsin	18,52	10	18	10	405	54,04	10
	Q12306	Ubiquitin-like protein SMT3	46,53	4	6	4	101	16,71	4
	P00729	Carboxypeptidase Y	8,83	7	9	7	532	14,28	7
	P43562	Probable metabolite transport protein YFL040W	2,41	1	1	1	540	2,31	1
	P40302	Proteasome subunit alpha type-6	7,26	1	1	1	234	2,27	1

P38776	Probable drug/proton antiporter YHK8	1,95	1	1	1	514	2,12	1
Q12691	Sodium transport ATPase 5	0,73	1	1	1	1091	1,98	1
Q01896	Sodium transport ATPase 2	0,73	1	1	1	1091	1,98	1
P13587	Sodium transport ATPase 1	0,73	1	1	1	1091	1,98	1
P38853	Kelch repeat-containing protein 1	1,03	1	1	1	1164	1,93	1

Table S1-5 The conditions of ultrahigh performance liquid chromatographic (UHPLC) separation and the settings for electrospray ionization-triple quadrupole-tandem mass spectrometry (ESI-QqQ-MS/MS) used for analysis of abscisic acid (ABA) in methanolic extracts of pea (*Pisum sativum* L., cultivar SGE) seeds with Waters ACQUITY UPLC H-Class UPLC System (Waters GmbH, Eschborn, Germany) coupled online to a hybrid triple quadrupole-linear ion trap mass spectrometer (QqLIT) AB Sciex QTRAP 6500 (AB Sciex, Darmstadt, Germany).

Chromatography

**ACQUITY Sample Manager (SM)**	
Injection mode	Partial Loop
Injection volume	5 µL
Weak wash solvent	0.3 mmol/L aq. ammonium formate
Weak wash volume	1200 µL
Strong wash solvent	Acetonitrile
Strong wash volume	600 µL
Target sample temperature	5.0 C
Needle overfill flush	Automatic
**Column conditions**	
Separation column	EC 150/2 NUCLEOSHELL RP 18<br>(150 x 2 mm, particle size 2.7 µm)
Target column temperature	40.0 C
**ACQUITY Binary Solvent Manager (BSM)**	
Eluent A	0.3 mmol/L aq. ammonium formate
Eluent B	Acetonitrile

Seal wash duration	5 min
Flow rate	0.4 mL/min
Elution program	5% eluent B isocratic – 2 min<br>gradient to 95% eluent B – 5.5 min<br>95% eluent B isocratic – 2 min<br>gradient to 5% eluent B – 0.01 min<br>5% eluent B isocratic – 1.99 min (re-equilibration)

Mass spectrometry

General	
Mass analyzer type	triple quadrupole-linear ion trap (QqLIT, QTRAP)
Ion source	Turbo Ion Spray®
Experiment type	multiple reaction monitoring (MRM)
Operatinon mode	negative
Cycle time (ms)	950
Pause between ranges (ms)	5.007
Settling time (s)	0
Duration	11 min
**Ion source settings**	
Nebulizer gas (psig)	60
Drying gas (psig)	70
Curtain gas (psig)	40
Ion spray voltage (kV)	-4.5
Ion source temperature (°C)	450

MS/MS Setting	
Fragmentation mode	CAD
MS/MS experiment type	MRM
Collision gas	nitrogen
Collision gas pressure	3 psig (medium)
Entrance potential (V)	-10.0
Scheduled MRM	enabled
Scheduled MRM type	basic
MRM detection window (s)	500
Target scan time (s)	1
Dwell time	adjusted by scheduled MRM algorithm
Q1 resolution	unit
Q3 resolution	unit
Declustering potential (DP, V)	compound-specific
Collision potential (CE, V)	compound-specific
Exit potential (CXP, V)	compound-specific

Analyte-specific settings

Analyte-specific combinations of Q1 and Q3 *m/z* ranges (transitions)						
**Analyte**	**t_R (min)**	**Q1 (*m/z*)**	**Q3 (*m/z*)**	**DP (V)**	**CE (V)**	**CXP (V)**
abscisic acid 01	4.8	263.0	152.9	-20.0	-16.0	-9.0
abscisic acid 02	4.8	263.0	219.0	-20.0	-18.0	-13.0
(d6) abscisic acid 01	4.8	269.0	159.0	-20.0	-16.0	-9.0
(d6) abscisic acid 02	4.8	269.0	225.0	-20.0	-18.0	-13.0

Table S1-6 Gas chromatographic (GC) separation conditions and electron ionization-quadrupole-mass spectrometry (EI-Q-MS) settings for analysis of *Pisum sativum* L. primary thermally stabile metabolites with Shimadzu GC2010 gas chromatograph coupled online to a quadrupole mass selective detector Shimadzu GCMS QP2010 with CTC GC PAL Liquid Injector (Shimadzu Scientific Instruments, Australia)

**Parameters**	**Setting**
	**GC settings**
Separation column	HP-5 capillary column (30 m × 0.25 mm ID, 0.25 μm film thickness, Thermo Fisher Scientific, Bremen, Germany)
Carrier gas / carrier gas flow rate	Helium/1 mL/min
Injector operation mode	Splitless mode (90 s splitless time)
Injector temperature	250°C
Temperature program	1 min at 40°C<br>ramp 15°C/min to 70°C<br>1 min at 70°C<br>ramp 6°C/min to 320°C<br>10 minat 320°C
**Parameters**	**MS settings**
Ionization mode	Electron ionization (EI)
Electron energy	70 eV
Operation mode	Positive, scanning at 0.34 sec $scan^{-1}$
m/z range	50 - 550

Table S1-7 The conditions of ion pair-reversed phase ultrahigh performance liquid chromatographic (IP-RP-UHPLC) separation and the settings for electrospray ionization-triple quadrupole-tandem mass spectrometry (ESI-QqQ-MS/MS) used for analysis of *Pisum sativum* L. anionic primary thermo labile metabolites with Waters ACQUITY UPLC H-Class UPLC System (Waters GmbH, Eschborn, Germany) coupled online to a hybrid triple quadrupole-linear ion trap mass spectrometer (QqLIT) AB Sciex QTRAP 6500 (AB Sciex, Darmstadt, Germany).

Chromatography

**ACQUITY Sample Manager (SM)**	
Injection mode	PartialLoop
Injection volume	5 µL
Weak wash solvent	0.3 mmol/L aq. ammonium formate
Weak wash volume	800 µL
Strong wash solvent	Acetonitrile
Strong wash volume	400 µL
Target sample temperature	4.0 C
Needle overfill flush	Automatic
**Column conditions**	
Separation column	EC 150/2 NUCLEOSHELL RP 18<br>(150 x 2 mm, particle size 2.7 µm)
Target column temperature	40.0 C
**ACQUITY Binary Solvent Manager (BSM)**	

Eluent A	0.3 mmol/L aq. ammonium formate
Eluent B	Acetonitrile
Seal wash duration	5 min
Flow rate	0.4 mL/min
Elution program	2% eluent B isocratic - 2 min<br>gradient to 36% eluent B – 16 min<br>gradient to 95% eluent B – 3 min<br>95% eluent B isocratic – 1.5 min<br>gradient to 2% eluent B – 0.1 min<br>2% eluent B isocratic – 2.4 min (re-equilibration)

Mass spectrometry

General	
Mass analyzer type	triple quadrupole-linear ion trap (QqLIT, QTRAP, operated in QqQ mode)
Ion source	TurboIonSpray®
Experiment type	multiple reaction monitoring (MRM)
Operatinon mode	negative
Cycle time (s)	1.1
Pause between ranges (ms)	5.007
Settling time (s)	0
Duration	24 min

Ion source settings	
Nebulizer gas (psig)	60
Drying gas (psig)	70

Curtain gas (psig)	40
Ion spray voltage (kV)	-4.5
Ion source temperature (°C)	450
**MS/MS settings**	
Fragmentation mode	CAD
MS/MS experiment type	MRM
Collision gas	nitrogen
Collision gas pressure	3 psig (medium)
Entrance potential (V)	-10.0
Scheduled MRM	enabled
Scheduled MRM type	basic
MRM detection window (s)	500
Target scan time (s)	1
Dwell time	adjusted by scheduled MRM algorithm
Q1 resolution	unit
Q3 resolution	unit
Declustering potential (DP, V)	compound-specific (listed below)
Collision potential (CE, V)	compound-specific (listed below)
Exit potential (CXP, V)	compound-specific (listed below)

Analyte-specific settings

Analyte-specific combinations of Q1 and Q3 *m/z* ranges (transitions)

#	Analyte	t_R (min)	Q1 (*m/z*)	Q3 (*m/z*)	DP (V)	CE (V)	CXP (V)
**1**	3-dehydroxyshikimic acid	0.0	171.0	127.0	-25.0	-16.0	-15.0
**2**	5-methyl-tetrahydrofolate	0.0	453.872	241.7	-20.0	-30.0	-15
**3**	allantoin	0.0	156.96	97.2	-60	-16	-1

**4**	3-ureidopropionic acid	0.0	130.9	87.9	-10	-14	-13
**5**	4-diphosphocytidyl-2-C-methyl-*D*-erythritol	0.0	520.1	78.9	-120	-108	-9
**6**	dehydroascorbic acid	0.0	173	127	-15	-18	-17
**7**	2-deoxyribose 5-phosphate	0.0	212.947	97.1	-40	-20	-19
**8**	flavin mononucleotide	0.0	498.814	480.9	-35	-18	-29
**9**	guanine	0.0	150.907	135.8	-10	-18	-9
**10**	γ-aminobutyric acid	0.0	100.944	56.9	-5	-12	-5
**11**	3-hydroxy-3-methylglutaric acid	0.0	161	99	-50	-35	-13
**12**	3-hydroxy-3-methylglutaric acid	0.0	161	57	-50	-45	-13
**13**	nicotinic acid ribonucleotide	0.0	333.885	289.9	-25	-16	-13
**14**	nicotinamide	0.0	120.933	76.9	-40	-16	-9
**15**	nicotinic acid	0.0	121.907	77.9	-55	-16	-13
**16**	beta-Nicotinamide mononucleotide	0.0	334.973	204.9	-50	-30	-13
**17**	quinic acid	0.0	190.932	85	-50	-28	-13
**18**	shikimic acid	0.0	172.892	92.9	-15	-20	-5
**19**	galactose-uridine-5'-diphosphate	0.0	564.805	322.8	-160	-32	-21
**20**	uridine-diphosphate-*N*-acetylglucosamine	0.0	605.754	384.8	-175	-36	-25
**21**	xanthopterin	0.0	178.833	107	-5	-12	-3
**22**	alanine	1.6	148	88.1	-20	-8	-3
**23**	arginine	1.6	173.2	131	-50	-18	-7
**24**	asparagine	1.6	131.1	87.1	-75	-16	-11
**25**	levocarnitine	1.6	220.2	145.9	-35	-12	-7
**26**	citrulline	1.6	174.2	131	-35	-18	-7
**27**	creatine	1.6	130.1	88.1	-25	-14	-5
**28**	cysteine/cystine	1.6	239.3	120	-40	-32	-1
**29**	glutamine	1.6	145.1	108.9	-30	-18	-5
**30**	glucose	1.6	178.9	89	-50	-12	-13
**31**	glycine	1.6	74.1	74	-36	-13	-3
**32**	lysine	1.6	145.2	101	-65	-14	-5
**33**	proline	1.6	114.1	86	-55	-18	-3

**34**	serine	1.6	104.1	74	-20	-16	-3
**35**	threonine	1.6	118.1	73.9	-25	-18	-3
**36**	ornithine	1.7	131.2	82.9	-60	-20	-5
**37**	sucrose	1.7	341.1	89.01	-240	-38	-13
**38**	valine	1.7	233.3	116	-25	-10	-5
**39**	cytidine	1.8	242.2	108.86	-70	-18	-5
**40**	histidine	1.8	154.2	93	-40	-24	-3
**41**	methionine	1.8	148.2	47	-45	-24	-5
**42**	aspartic acid	1.85	132	88	-40	-18	-13
**43**	leucine + isoleucine	1.9	261.3	130.2	-30	-10	-1
**44**	tyrosine	1.9	180.2	118.9	-60	-24	-5
**45**	uridine	1.9	243.2	109.88	-65	-22	-5
**46**	glucosamine 6-phosphate	2.1	258.2	97	-45	-24	-5
**47**	glutamic acid	2.1	146	102	-80	-18	-9
**48**	guanosine	2.1	282.2	149.93	-80	-26	-7
**49**	phosphocholine	2.3	242.2	167.9	-40	-12	-9
**50**	phenylalanine	2.4	164.2	103	-55	-24	-5
**51**	S-adenosyl-*L*-homocysteine	2.5	383.4	133.9	-80	-36	-7
**52**	adenosine	2.7	266.2	133.86	-70	-12	-1
**53**	argininosuccinic acid	2.7	289.3	271.3	-55	-14	-15
**54**	glucosamine 1-phosphate	2.7	258.2	78.9	-55	-42	-1
**55**	ascorbic acid	3.2	175	115	-25	-25	-5
**56**	tryptophan	3.5	203.2	116.2	-50	-22	-7
**57**	dihydroorotic acid	3.8	157.1	112.7	-40	-10	-5
**58**	lactic acid	3.8	89.1	42.9	-15	-12	-5
**59**	phosphate	4.6	96.9	78.9	-40	-18	-15
**60**	glutathione	4.7	306.3	143	-5	-26	-7
**61**	orotic acid	4.8	155.1	110.7	-25	-12	-5
**62**	pyruvic acid	4.9	87.1	43	-30	-12	-1
**63**	mevalonic acid lactone	5.1	147.2	59.1	-45	-20	-7
**64**	nicotinamide adenine dinucleotide	5.5	662.4	540.1	-45	-22	-15

**65**	glucose 6-phosphate	5.9	259.1	97	-65	-18	-13
**66**	erythrose 4-phosphate	6.09	199.1	96.8	-40	-12	-5
**67**	2-deoxyribose 5-phosphate	6.1	229.1	96.81	-35	-20	-5
**68**	fructose 6-phosphate	6.3	259.1	96.9	-30	-20	-11
**69**	glycerophosphoric acid	6.3	171.1	78.8	-45	-24	-1
**70**	sedoheptulose 7-phosphate	6.3	289.2	97	-50	-22	-5
**71**	cyclic guanosine monophosphate	6.4	344.2	150	-70	-34	-11
**72**	glyceraldehyde 3-phosphate	6.5	169	97.01	-30	-12	-5
**73**	glucose-1-phosphate	6.7	259.1	240.8	-30	-16	-15
**74**	2-C-methylerythritol 4-phosphate	6.86	215	78.9	-40	-56	-9
**75**	cytidine monophosphate	7	322.2	79	-65	-68	-5
**76**	pantothenic acid	7	218.2	88.1	-55	-18	-5
**77**	adenosine 3',5'-cyclic mono-phosphate	7.6	328.2	133.9	-125	-36	-5
**78**	ribulose-5-phosphate/xylulose-5-phosphate	7.6	229.1	96.8	-45	-18	-15
**79**	ribose-1-phosphate	7.7	229.1	211	-50	-14	-3
**80**	guanosine 5'-monophosphate	7.8	362.2	78.92	-65	-66	-5
**81**	uridine monophosphate	7.8	323.2	79	-65	-68	-5
**82**	5-amino-4-imidazolecarboxamide ribotide	8.1	337.2	79.1	-85	-50	-5
**83**	inosinic acid	8.1	347.2	134.8	-70	-38	-7
**84**	2'-deoxyguanosine 5'-monophosphate	8.4	346.2	78.81	-80	-42	-3
**85**	dihydroxyacetone phosphate	8.47	169.1	97	-35	-14	-11
**86**	1-deoxy-*D*-xylulose 5-phosphate	8.6	213.129	97	-50	-18	-1
**87**	thymidine-5'-phosphate	8.7	321.2	78.81	-65	-58	-3
**88**	adenosine monophosphate	8.9	346.2	78.82	-70	-52	-3
**89**	glutathione disulfide	9.5	611.6	306.1	-35	-34	-7
**90**	phosphocreatine	9.6	210.1	78.9	-35	-22	-1
**91**	malate	9.9	133.1	115	-20	-16	-5
**92**	succinic acid	9.9	117.11	73	-25	-16	-7
**93**	adenosine diphosphoribose	10	557.94	346	-170	-34	-19

**94**	ureidosuccinic acid	10	175.1	131.8	-25	-16	-7
**95**	adenosine diphosphate glucose	10.2	587.864	345.9	-140	-32	-19
**96**	sulfate	10.2	97	97	-40	-18	-15
**97**	uridine-5'-diphosphate-glucose	10.2	565.3	323	-125	-36	-11
**98**	fumaric acid	10.6	115.101	71	-5	-12	-13
**99**	α-ketoglutaric acid	10.7	145.01	101	-10	-12	-13
**100**	2C-methyl-*D*-erythritol 2,4-cyclodiphosphate	10.9	277	79	-45	-64	-37
**101**	1,4-dihydronicotinamide adenine dinucleotide	11.4	664.4	78.9	-100	-124	-1
**102**	(R)-5-phosphomevalonic acid	12.6	227.1	97	-30	-35	-10
**103**	cytidine-5'-diphosphate	12.8	402.2	78.87	-65	-70	-5
**104**	2-phosphoglyceric acid	12.9	185	79.01	-25	-20	-35
**105**	guanosine-5'-diphosphate	12.9	442.2	78.85	-85	-70	-3
**106**	xanthosine-5'-phosphate	12.9	363.2	151.1	-60	-36	-5
**107**	6-phosphogluconic acid	13.1	275	79	-60	-66	-5
**108**	thymidine-5'-diphosphate	13.1	401.2	78.81	-70	-68	-3
**109**	uridine-5'-diphosphate	13.1	403.2	78.79	-75	-68	-3
**110**	3-phosphoglyceric acid	13.2	185.1	96.7	-30	-22	-7
**111**	adenosine-5'-diphosphate	13.2	426.2	78.85	-75	-66	-3
**112**	2'-deoxyadenosine-5'-diphosphate	13.3	410.2	78.88	-60	-76	-3
**113**	(2E)-4-hydroxy-3-methylbut-2-en-1-yl diphosphate	13.3	261	79	-40	-52	-9
**114**	aconitic acid	13.4	172.866	128.7	-25	-10	-55
**115**	flavin adenine dinucleotide	13.4	784.5	79	-60	-130	-1
**116**	nicotinamide adenine dinucleotide phosphate	13.4	742.4	620	-55	-22	-17
**117**	phosphoenolpyruvic acid	13.86	167	78.8	-20	-16	-9
**118**	citric acid	14.1	191	87	-35	-22	-15
**119**	isocitric acid	14.39	191.1	73	-45	-28	-31
**120**	3'-dephospho coenzyme A	14.5	686.6	78.8	-105	-112	-1
**121**	isopentenyl pyrophosphate	14.6	245.03	78.9	-15	-44	-37
**122**	orotidine 5'-monophosphate	14.9	390.2	78.9	-50	-78	-1

**123**	fructose-1,6-diphosphate	15.1	339.1	96.9	-35	-22	-11
**124**	cytidine 5'-triphosphate	15.4	482.2	158.83	-85	-36	-9
**125**	deoxythymidine 5'-triphosphate	15.4	481.2	158.71	-80	-38	-9
**126**	guanosine-5'-triphosphate	15.4	522.2	158.79	-90	-48	-9
**127**	uridine-5'-triphosphate	15.5	483.1	158.76	-90	-38	-9
**128**	adenosine triphosphate	15.6	506.2	158.78	-80	-38	-9
**129**	ribulose-1,5-bisphosphate	15.7	309.1	97	-35	-20	-27
**130**	adenylosuccinic acid	15.8	462.3	133.9	-85	-62	-7
**131**	mevalonate-5-diphosphate	15.9	307	78.9	-25	-35	-13
**132**	4-diphosphocytidyl-2-C-methyl-*D*-erythritol 2-phosphate	16	600	78.9	-115	-126	-19
**133**	sedoheptulose-7-phosphate	16	369	97	-35	-20	-27
**134**	dihydronicotinamide adenine dinucleotide phosphate	16.3	744.4	79	-110	-118	-3
**135**	coenzyme A	16.8	766.5	78.9	-40	-122	-1
**136**	5-phosphoribosyl diphosphate	16.8	389.1	176.8	-55	-28	-9
**137**	inositol triphosphate	17.4	418.737	320.8	-25	-28	-21
**138**	acetoacetyl coenzyme A	17.5	424.8	382.6	-50	-12	-11
**139**	coenzyme A	17.5	765.8	407.9	-245	-50	-19
**140**	propyonyl-CoA	17.6	822.6	78.8	-120	-130	-13
**141**	S-acetyl coenzyme A	17.7	807.9	407.9	-220	-52	-27
**142**	methylmalonyl coenzyme A	17.8	432.8	410.6	-25	-8	-13
**143**	butanoyl coenzyme A	17.9	417.8	78.84	-50	-74	-3
**144**	geranyl diphosphate	18	313.2	78.9	-65	-46	-1
**145**	inositol-1,3,4,5-tetraphosphate	18.2	498.704	400.7	-100	-30	-27
**146**	malonyl coenzyme A	18.6	851.9	408	-20	-8	-11
**147**	malonyl coenzyme A	18.6	851.9	807.9	-185	-36	-37
**148**	succinyl coenzyme A	18.6	866	407.6	-260	-56	-25
**149**	β-methylcrotonyl coenzyme A	19.1	847.9	407.8	-240	-58	-23
**150**	hydroxymethylglutaroyl coenzyme A	19.1	454.8	382.6	-50	-18	-9
**151**	1-diphosinositol pentakisphosphate	19.2	578.755	480.6	-25	-32	-31

**152**	isovaleryl coenzyme A	19.2	849.9	407.9	-240	-58	-19
**153**	geranylgeranyl pyrophosphate	19.6	449.069	78.8	-65	-68	-35
**154**	Phytic acid	19.8	658.555	560.7	-145	-38	-31
**155**	geranylfarnesyl diphosphate	20.4	517.188	78.8	-45	-84	-19
**156**	ent-copal-8-ol diphosphate	21	467.18	78.8	-240	-38	-13
**157**	farnesyl diphosphate	21.2	381.3	78.9	-50	-50	-5

Table S1-8 The conditions of ultrahigh performance liquid chromatographic (UHPLC) separation and the settings for electrospray ionization-quadrupole-time of flight mass spectrometry (ESI-QqTOF-MS) applied for the analysis of *Pisum sativum* L. semi-polar secondary metabolites with Waters ACQUITY UPLC I-Class UPLC System (Waters GmbH, Eschborn, Germany) coupled online to a hybrid quadrupole-time of flight mass spectrometer (QqTOF-MS) AB Sciex TripleTOF 6600 (AB Sciex, Darmstadt, Germany).

Chromatography

**ACQUITY Sample Manager (SM)**	
Injection mode	Partial Loop
Injection volume	5 µL
Weak wash solvent	0.3 mmol/L aq. ammonium formate
Weak wash volume	800 µL
Strong wash solvent	acetonitrile
Strong wash volume	400 µL
Target sample temperature	4.0 C
Needle overfill flush	automatic

Column conditions

Separation column	EC 150/2 NUCLEOSHELL RP 18<br>(150 x 2 mm, particle size 2.7 µm)
Target column temperature	40.0 C

ACQUITY Binary Solvent Manager (BSM)	
Eluent A	0.3 mmol/L aq. ammonium formate
Eluent B	acetonitrile
Seal wash duration	5 min
Flow rate	0.4 mL/min
Elution program	5% eluent B isocratic - 2 min<br>gradient to 95% eluent B – 17 min<br>95% eluent B isocratic – 2 min<br>gradient to 5% eluent B – 0.1 min<br>5% eluent B isocratic – 3 min (re-equilibration)

Mass spectrometry

General	
Mass analyzer type	quadrupole-time of flight (QqTOF-MS)
Ionsource	DuoSpray™ ion source
Experiment type	Sequential Windowed Acquisition of All Theoretical Fragment Ion Mass Spectra (SWATH)
Operatinon mode	positive/negative
Cycle time (s)	1.1
Pause between ranges (ms)	1.049
Auto adjust with mass	on

Settling time (s)	0
Time bins to sum	4
Duration	23 min
	**Ion source settings**
Nebulizer gas (psig)	60
Drying gas (psig)	70
Curtain gas (psig)	55
Ion spray voltage (kV)	5.5/-4.5 (positive/negative mode)
Ion source temperature (°C)	450
	**MS settings**
Experiment type	TOF-MS
m/z range	65 - 1250
Accumulation time (ms)	100
Declustering potential (V)	35/-35 (positive/negative mode)
Collision potential (V)	10/-10 (positive/negative mode)
	**MS/MS Setting**
Fragmentation mode	collision-activated dissociation (CAD)3
MS/MS experiment type	SWATH
SWATH window number	48
SWATH window width (*m/z*)	26
SWATH window overlap (*m/z*)	1
Rolling collision energy	off
Analyte type	small molecules
Accumulation time (ms)	20
Declustering potential (V)	35/-35 (positive/negative mode)

Collision potential (V)	45/-35 (positive/negative mode)
Collision energy spread (V)	35/15 (positive/negative mode)
Ion relese delay (V)	30/-30 (positive/negative mode)
Ion release width (V)	15/-15 (positive/negative mode)

Table S1-9 Composition of the alkane mixture used for determination of Kovats retention time indices (RIs)

Alkane name	Elemental composition	RI	Ref. *m/z*	t_R (min)
Dodecane	$C_{12}H_{26}$	1200	170	12.71
Tridecane	$C_{13}H_{28}$	1300	184	15.025
Tetradecane	$C_{14}H_{30}$	1400	198	17.252
Pentadecane	$C_{15}H_{32}$	1500	212	19.349
Hexadecane	$C_{16}H_{34}$	1600	226	21.335
Heptadecane	$C_{17}H_{36}$	1700	240	23.217
Octadecane	$C_{18}H_{38}$	1800	254	25.002
Nonadecane	$C_{19}H_{40}$	1900	268	26.709
Eicosane (= Icosane)	$C_{20}H_{42}$	2000	282	28.336
Heneicosane	$C_{21}H_{44}$	2100	296	29.891
Docosane	$C_{22}H_{46}$	2200	310	31.384
Tricosane	$C_{23}H_{48}$	2300	324	32.817
Tetracosane	$C_{24}H_{50}$	2400	338	34.194
Pentacosane	$C_{25}H_{52}$	2500	352	35.52
Hexacosane	$C_{26}H_{54}$	2600	366	36.793
Heptacosane	$C_{27}H_{56}$	2700	380	38.016
Octacosane	$C_{28}H_{58}$	2800	394	39.211
Nonacosane	$C_{29}H_{60}$	2900	408	40.36
Triacontane	$C_{30}H_{62}$	3000	422	41.472
Hentriacontane	$C_{31}H_{64}$	3100	436	42.547
Dotriacontane	$C_{32}H_{66}$	3200	450	43.59
Tritriacontane	$C_{33}H_{68}$	3300	464	44.603
Tetratriacontane	$C_{34}H_{70}$	3400	478	45.587
Pentatriacontane	$C_{35}H_{72}$	3500	492	46.599
Hexatriacontane	$C_{36}H_{74}$	3600	506	47.736
Heptatriacontane	$C_{37}H_{76}$	3700	520	49.039
Octatriacontane	$C_{38}H_{78}$	3800	534	50.544
Nonatriacontane	$C_{39}H_{80}$	3900	548	52.304
Tetracontane	$C_{40}H_{82}$	4000	562	55.001

Figures

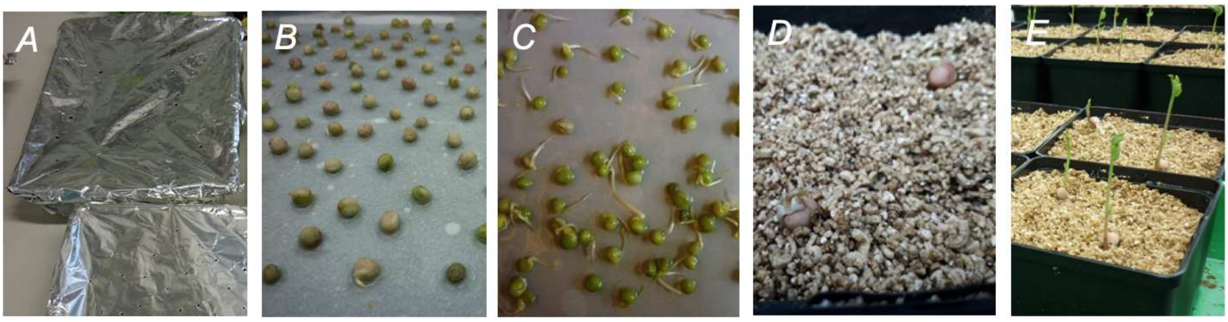

Figure S1-1 Cultivation of pea (*Pisum sativum* L., cultivar SGE) plants: stratification on wet filter paper in the dark at 4°C during two days (A), germination during two days in the dark (B, C), inoculation with a rhizobial culture *Rhizobium leguminosarum*1026 ICAM (D), and seedling appearance – growing at 16 h light/ 8 h dark regimen at 21°C under 75% relative humidity (G).

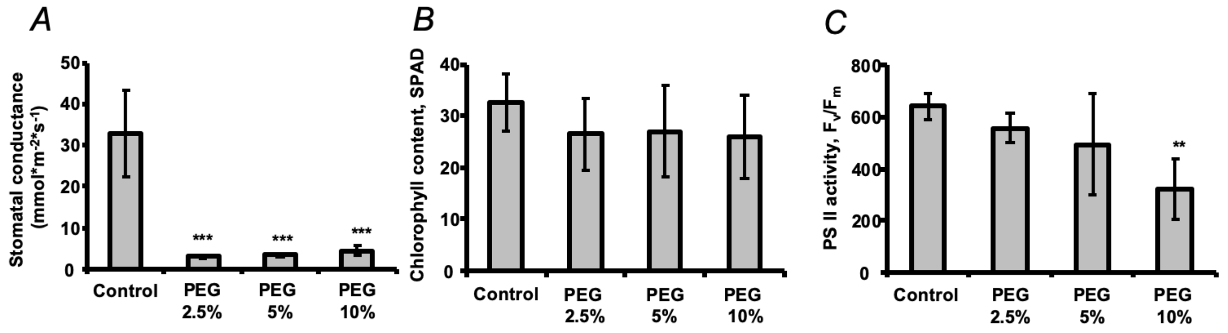

Figure S1-2 Optimization of experimental drought conditions applied to pea plants pea (*Pisum sativum* L., cultivar SGE) plants at the stage of seed maturation. The plants were transferred from vermiculite to an aqueous aerated medium on the 41st day after inoculation (d.a.i.), and five days later (on the 46th d.a.i.) - to aqueous medium saturated with PEG-free (control) or PEG 8000 (osmotic stress) at the concentration of 2.5, 5, or 10% (w / v) solutions. After two days of exposure to osmotic stress, the roots of the plants were washed and the plants were transferred to vermiculite. The physiological parameters — stomatal conductivity (A), chlorophyll content (B) and photosystem II (PS II) efficiency (C), were assessed three days later, before the plants were transferred to PEG-free medium.

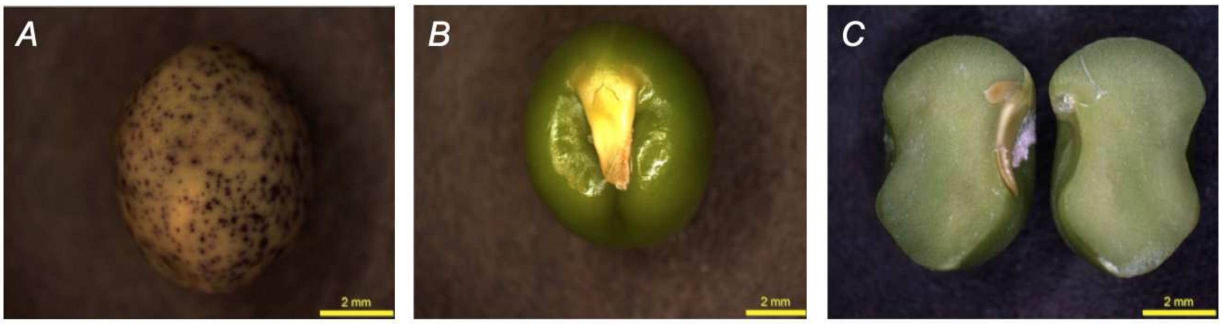

Figure S1-3 Morphology and anatomy of pea (*Pisum sativum* L., cultivar SGE) seeds: appearance of the seed (A), seed embryo (B) and its longitudinal section (C).

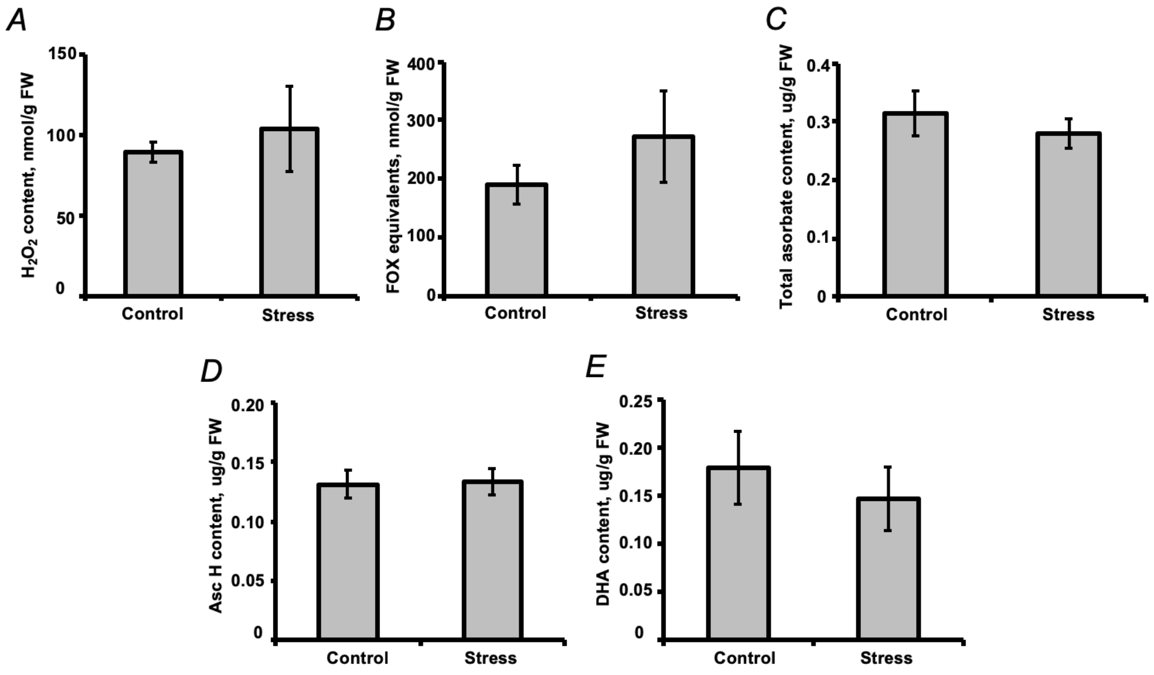

Figure S1-4 Characterization of the stress response in pea (*Pisum sativum* L., cultivar SGE) leaves, observed after a two-day exposure of mature plants (at the seed maturation step) to the aqueous medium with and without addition of 2.5% (w/v) PEG 8000 (defined as Stress and Control, respectively) by the contents of hydrogen peroxide (A), lipid hydroperoxides (as 13*S*-hydroperoxy-9*Z*, 11*E*-octadecanoic acid equivalents, B), total ascorbate (C), ascorbic acid (D) and dehydroascorbate (E).

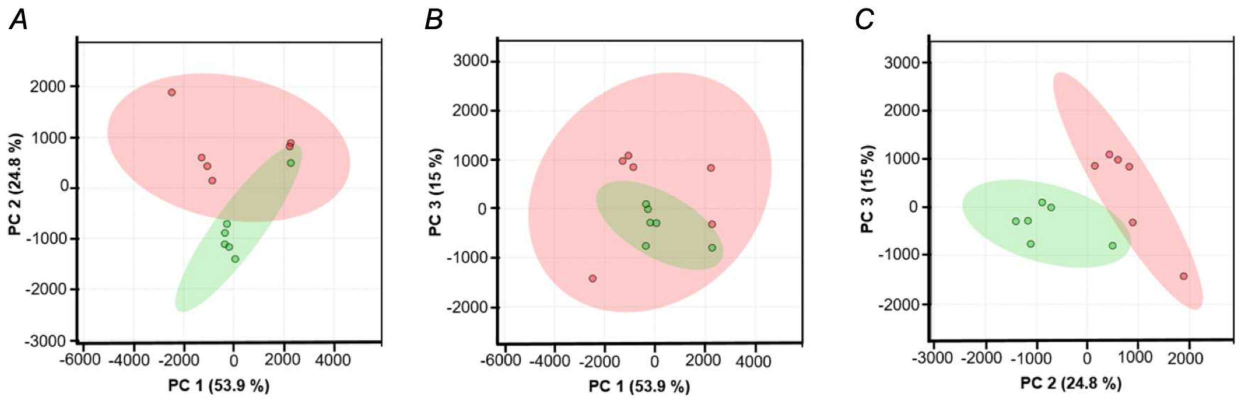

Figure S1-5 The results of the principal component analysis (PCA), done for the abundances of the primary thermally stabile polar metabolites, detected by untargeted gas chromatography-electron ionization-quadrupole mass spectrometry (GC-EI-Q-MS) in aqueous methanolic extracts of mature pea (*Pisum sativum* L., cultivar SGE) seeds after a two-day exposure of mature plants (at the seed maturation step) to the aqueous medium with (green) and without (red) addition of 2.5% (w/v) PEG 8000 (defined as Stress and Control, respectively). The score plots represent PC1 plotted against PC2 (A), PC1 plotted against PC3 (B) and PC2 plotted against PC3 (C).

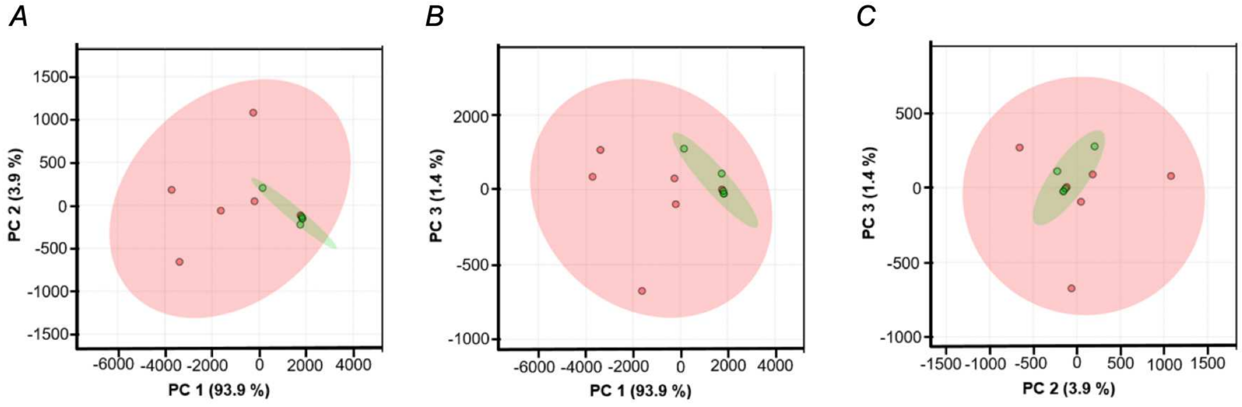

Figure S1-6 The results of the principal component analysis (PCA), done for the abundances of the primary thermally labile anionic polar metabolites, detected by targeted ion pair-reversed phase ultrahigh performance liquid chromatography, coupled on-line to electrospray ionization-triple quadrupole tandem mass spectrometry (IP-RP-UHPLC-ESI-QqQ-MS/MS) in acidified ethanol-aqueous extracts of mature pea (*Pisum sativum* L., cultivar SGE) seeds after a two-day exposure of mature plants (at the seed maturation step) to the aqueous medium with (green) and without (red) addition of 2.5% (w/v) PEG 8000 (defined as Stress and Control, respectively). The score plots represent PC1 plotted against PC2 (A), PC1 plotted against PC3 (B) and PC2 plotted against PC3 (C).

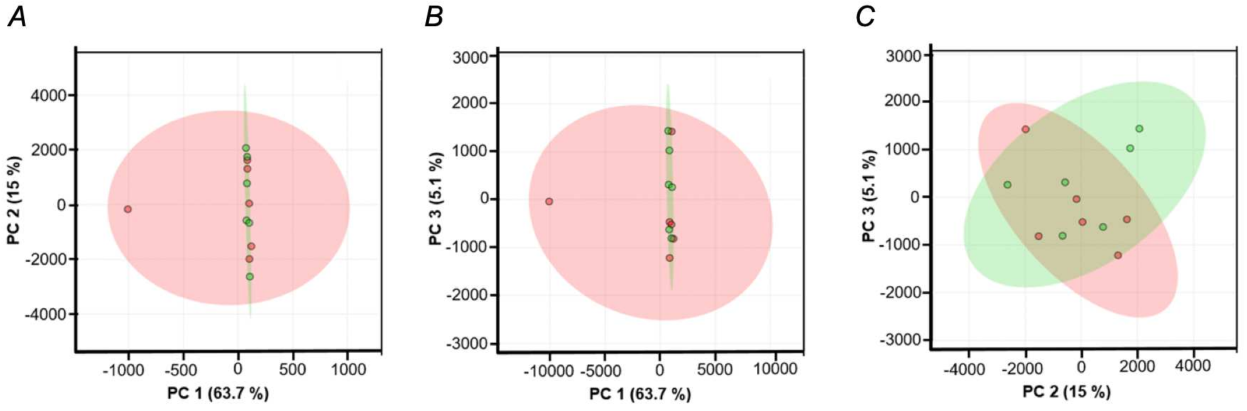

Figure S1-7 The results of the principal component analysis (PCA), done for the abundances of the anionic semi-polar secondary metabolites, detected by untargeted reversed phase ultrahigh performance liquid chromatography, coupled on-line to electrospray ionization-quadrupole time-of-flight (tandem) mass spectrometry (RP-UHPLC-ESI-QqTOF-MS and MS/MS operated in negative ion mode) in ethanol-dichloromethane extracts of mature pea (*Pisum sativum* L., cultivar SGE) seeds after a two-day exposure of mature plants (at the seed maturation step) to the aqueous medium with (green) and without (red) addition of 2.5% (w/v) PEG 8000 (defined as Stress and Control, respectively). The MS/MS analyses were designed as sequential window acquisition of all theoretical fragment ion spectra (SWATH) experiments. The score plots represent PC1 plotted against PC2 (A), PC1 plotted against PC3 (B) and PC2 plotted against PC3 (C).

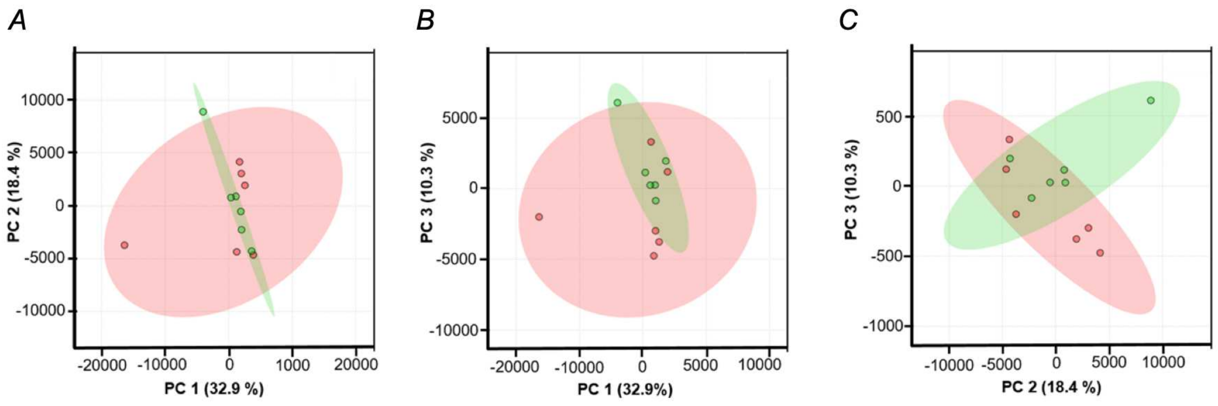

Figure S1-8 The results of the principal component analysis (PCA), done for the abundances of the cationic semi-polar secondary metabolites, detected by untargeted reversed phase ultrahigh performance liquid chromatography, coupled on-line to electrospray ionization-quadrupole time-of-flight (tandem) mass spectrometry (RP-UHPLC-ESI-QqTOF-MS and MS/MS operated in positive ion mode) in ethanol-dichloromethane extracts of mature pea (*Pisum sativum* L., cultivar SGE) seeds after a two-day exposure of mature plants (at the seed maturation step) to the aqueous medium with (green) and without (red) addition of 2.5% (w/v) PEG 8000 (defined as Stress and Control, respectively). The MS/MS analyses were designed as sequential window acquisition of all theoretical fragment ion spectra (SWATH) experiments. The score plots represent PC1 plotted against PC2 (A), PC1 plotted against PC3 (B) and PC2 plotted against PC3 (C).

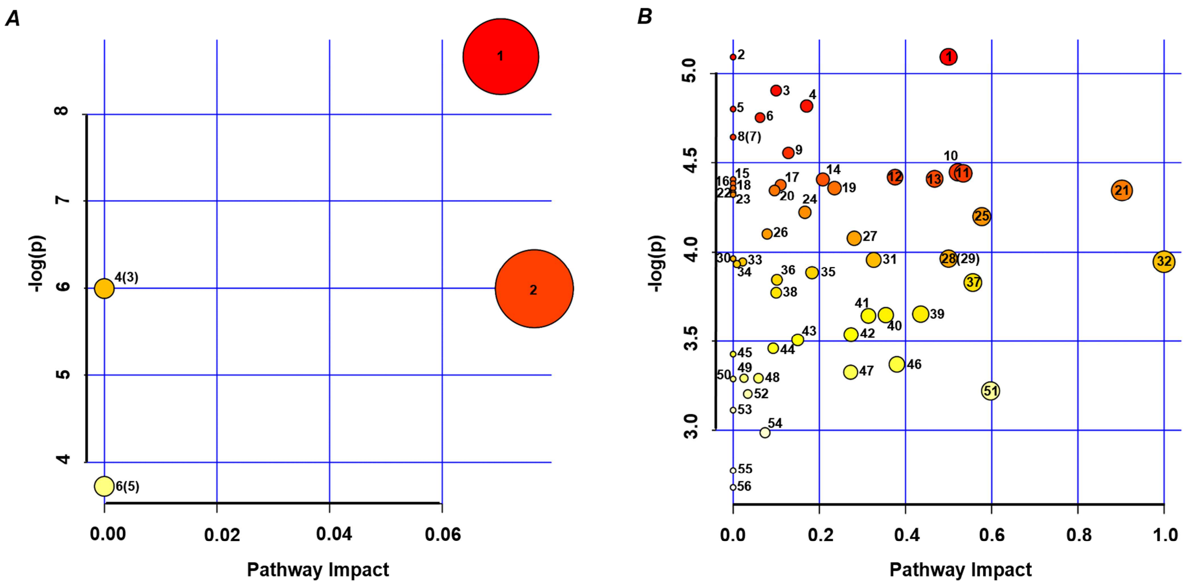

Figure S1-9 Pathway analyses accomplished separately for the drought-regulated (more than 1.5-fold, t-test p < 0.05, FDR adjusted at p < 0.1) primary polar metabolites of pea (*Pisum sativum* L., cultivar SGE) seeds annotated by untargeted GC-EI-Q-MS (A) and targeted IP-RP-UHPLC-MS/MS (B). ***A***: 1, galactose metabolism; 2, glycine, serine and threonine metabolism; 3, cysteine and methionine metabolism; 4, lysine biosynthesis; 5, fructose and mannose metabolism; 6, amino sugar and nucleotide sugar metabolism. ***B***: 1, isoquinoline alkaloid biosynthesis; 2, ubiquinone and other terpenoid-quinone biosynthesis; 3, phenylalanine, tyrosine and tryptophan biosynthesis; 4, tryptophan metabolism; 5, indole alkaloid biosynthesis; 6, histidine metabolism; 7, sphingolipid metabolism; 8, sulfur metabolism; 9, glycerophospholipid metabolism; 10, pyrimidine metabolism; 11, glycine, serine and threonine metabolism; 12, arginine and proline metabolism; 13, nicotinate and nicotinamide metabolism; 14, β-alanine metabolism; 15, cyanoamino acid metabolism; 16, nitrogen metabolism; 17, carbon fixation in photosynthetic organisms; 18, porphyrin and chlorophyll metabolism; 19, butanoate metabolism; 20, cysteine and methionine metabolism; 21, alanine, aspartate and glutamate metabolism; 22, glucosinolate biosynthesis; 23, folate biosynthesis; 24, methane metabolism; 25, glutathione metabolism; 26, glycerolipid metabolism; 27, terpenoid backbone biosynthesis; 28, phenylalanine metabolism; 29, phenylpropanoid biosynthesis; 30, tropane, piperidine and

pyridine alkaloid biosynthesis; 31, purine metabolism; 32, synthesis and degradation of ketone bodies; 33, valine, leucine and isoleucine degradation; 34, zeatin biosynthesis; 35, ascorbate and aldarate metabolism; 36, galactose metabolism; 37, starch and sucrose metabolism; 38, pentose and glucuronate interconversions; 39, glyoxylate and dicarboxylate metabolism; 40, citrate cycle (TCA cycle); 41, fatty acid metabolism; 42, pyruvate metabolism; 43, pantothenate and CoA biosynthesis; 44, aminoacyl-tRNA biosynthesis; 45, inositol phosphate metabolism; 46, amino sugar and nucleotide sugar metabolism; 47, tyrosine metabolism; 48, propanoate metabolism; 49, fatty acid biosynthesis; 50, riboflavin metabolism; 51, pentose phosphate pathway; 52, glycolysis or Gluconeogenesis; 53, vitamin B6 metabolism; 54, lysine biosynthesis; 55, valine, leucine and isoleucine biosynthesis; 56, lysine degradation. For more information, please refer to Supplementary 4 and 5.

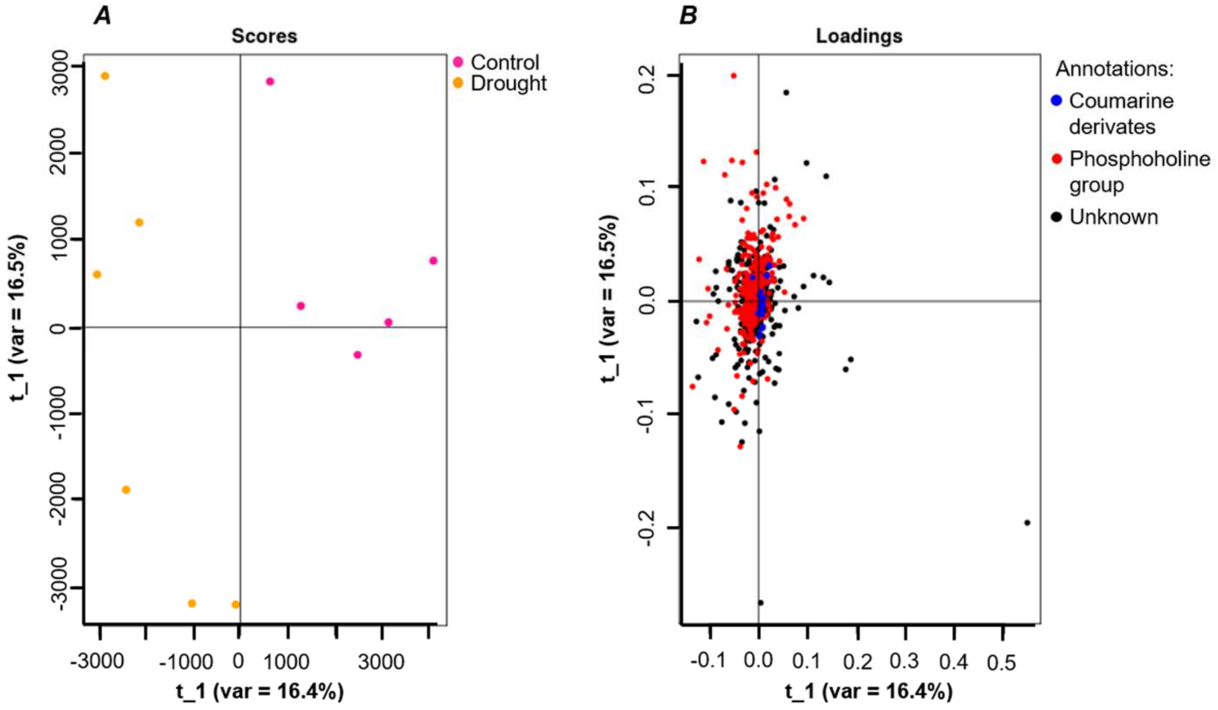

Figure S1-10 The PLS-DA model (*A*) and loadings plot (*B*), designed for 1092 of 2667 semi-polar features annotated by RP-UHPLC-QqTOF-MS operated in positive SWATH mode. The original data matrix was filtered for the presence of isotopes (43 features) and for the MS signal not accompanied with adequate MS/MS spectra (1532 features). Characteristic *m/z* of coumarine derivates: 91.0541, 102.0488, 104.0574, 115.0532, 137.0966, 163.0395; and of phosphoholine group – 184.0737. Annotation of fragments (± 10 ppm) and building of the PLS-DA model relied on the online tool MetFamily 1.0 tool (https://msbi.ipb-halle.de/MetFamilyDevel/).

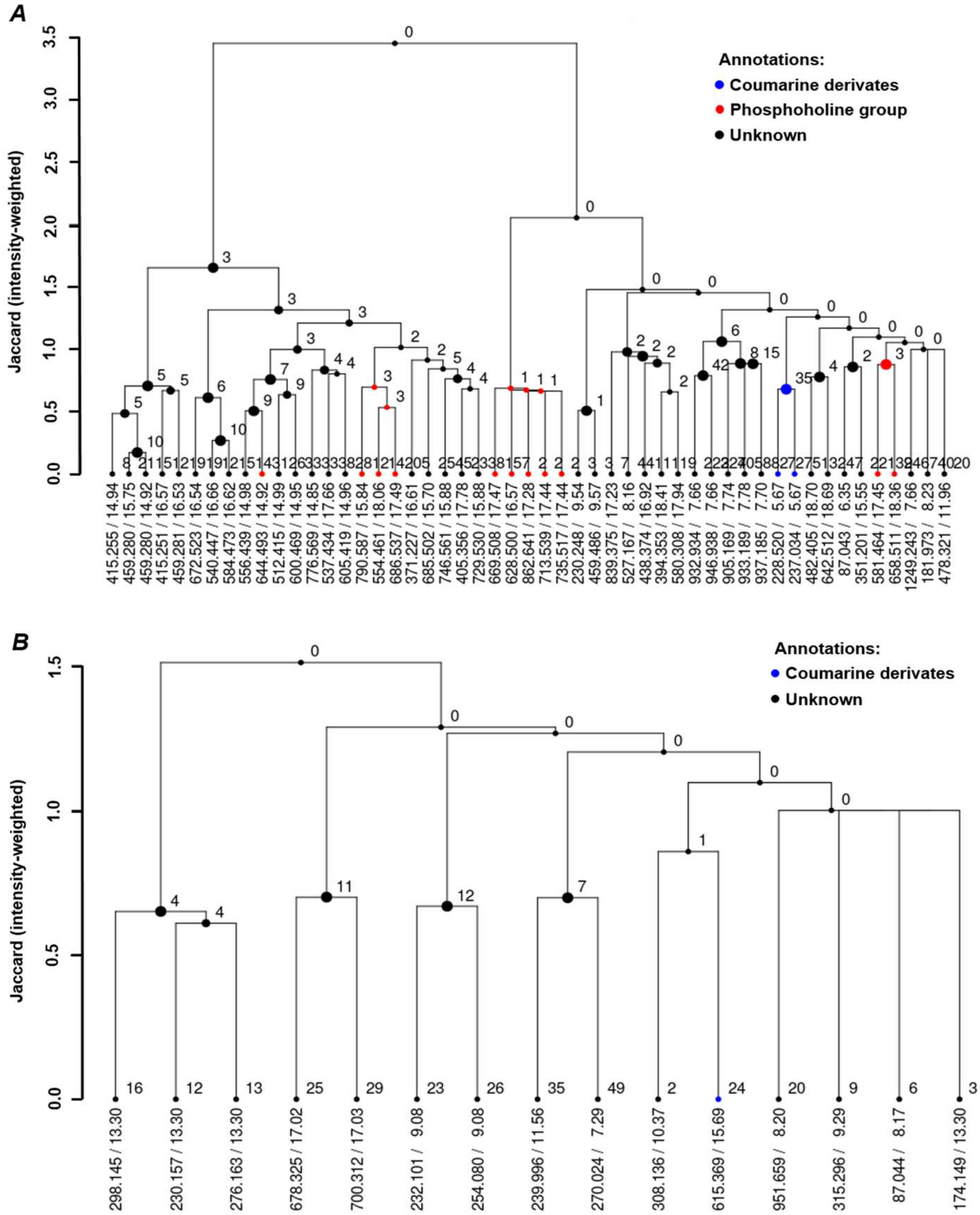

Figure S1-11 Hierarchical clustering analysis (HCA) of 51 up-regulated (*A*) and 15 down-regulated (*B*) drought-related semi-polar metabolite MS features using the corresponding MS/MS spectra obtained from RP-UHPLC-QqTOF-MS operated in positive SWATH mode. For the intergroup (stress-control) comparisons, the set of 1092 MS^1 features was filtered using an MS^1 abundance threshold of 2000 counts and a log2-fold change (LFC) of 0.58. Ion fragment search (± 10 ppm) and HCA was performed by the online tool MetFamily 1.0 (https://msbi.ipb-halle.de/MetFamilyDevel).

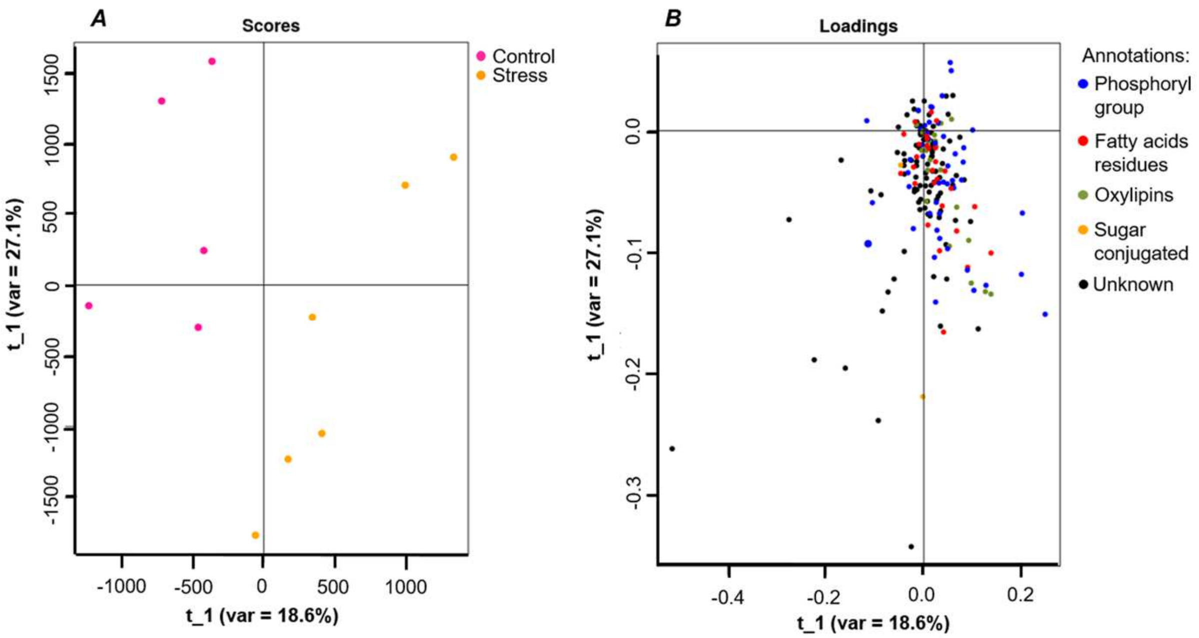

Figure S1-12 The PLS-DA model (*A*) and loadings plot (*B*), designed for 207 of 376 semi-polar metabolites identified by RP-UHPLC-QqTOF-MS operated in negative SWATH mode. The original set (376 features) was filtered for isotopomers and the features without interpretable MS/MS spectra (in total 166 features). The metabolite class of phospholipids was annotated by characteristic fragment signals (*m/z*) of phosphoryl group: 78.9585, 78.9591, 140.0118, 152.996, 168.0431, 171.0064, 196.038 and characteristic fragments (*m/z*) of fatty acid residues: 171.1391, 171.1398 for C10:0, 255.23303 for C16:0 and 279.233 for C18:2. Characteristic *m/z* of oxylipins: 183.01; of sugar conjugated metabolites: -303.098370, -323.098, -179.056. Ion fragment search (± 10 ppm) and PLS-DA model was performed by the online tool MetFamily 1.0 (https://msbi.ipb-halle.de/MetFamilyDevel).

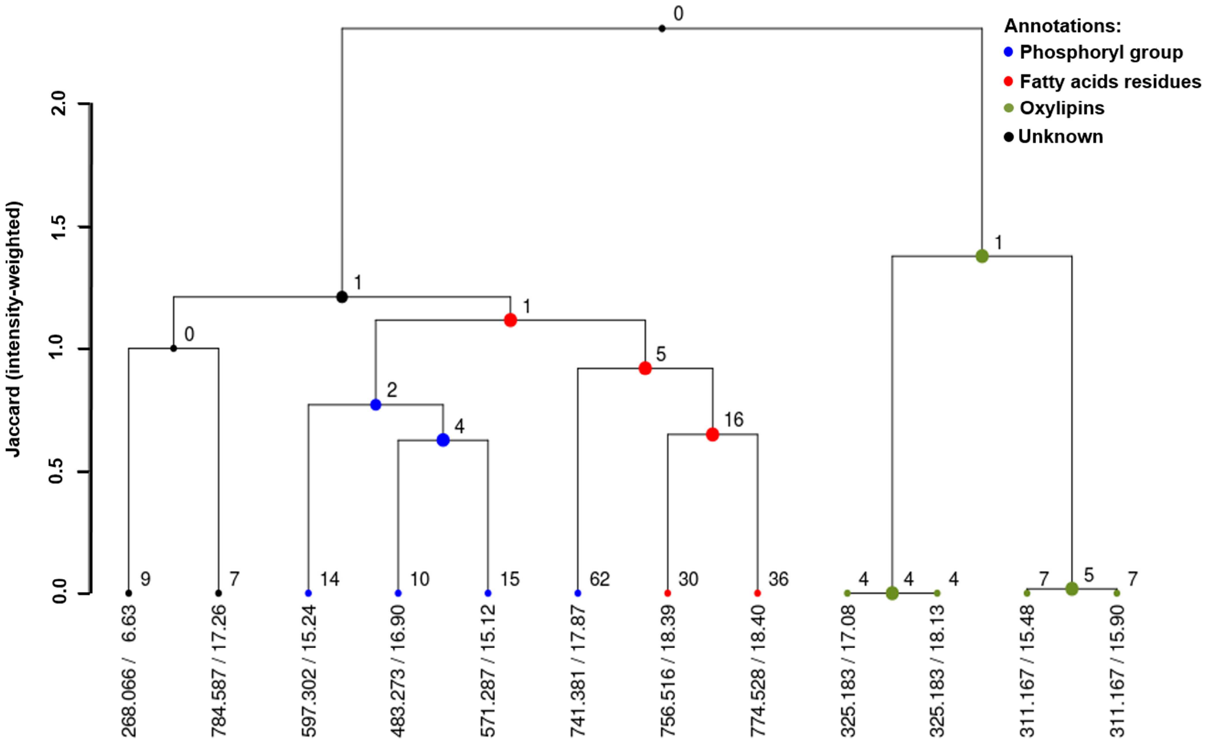

Figure S1-13 Hierarchical cluster analysis (HCA) of 12 up-regulated drought-related semi-polar metabolites (MS^1) using the corresponding MS/MS spectra obtained from RP-UHPLC-QqTOF-MS operated in negative SWATH mode. For comparison of the control versus stress groups the set of 207 MS^1 features was filtered using an MS^1 abundance threshold of 2000 counts, a log2-fold change (LFC) of 0.58. Ion fragment search (± 10 ppm) and HCA was performed by the online tool MetFamily 1.0 (https://msbi.ipb-halle.de/MetFamilyDevel).

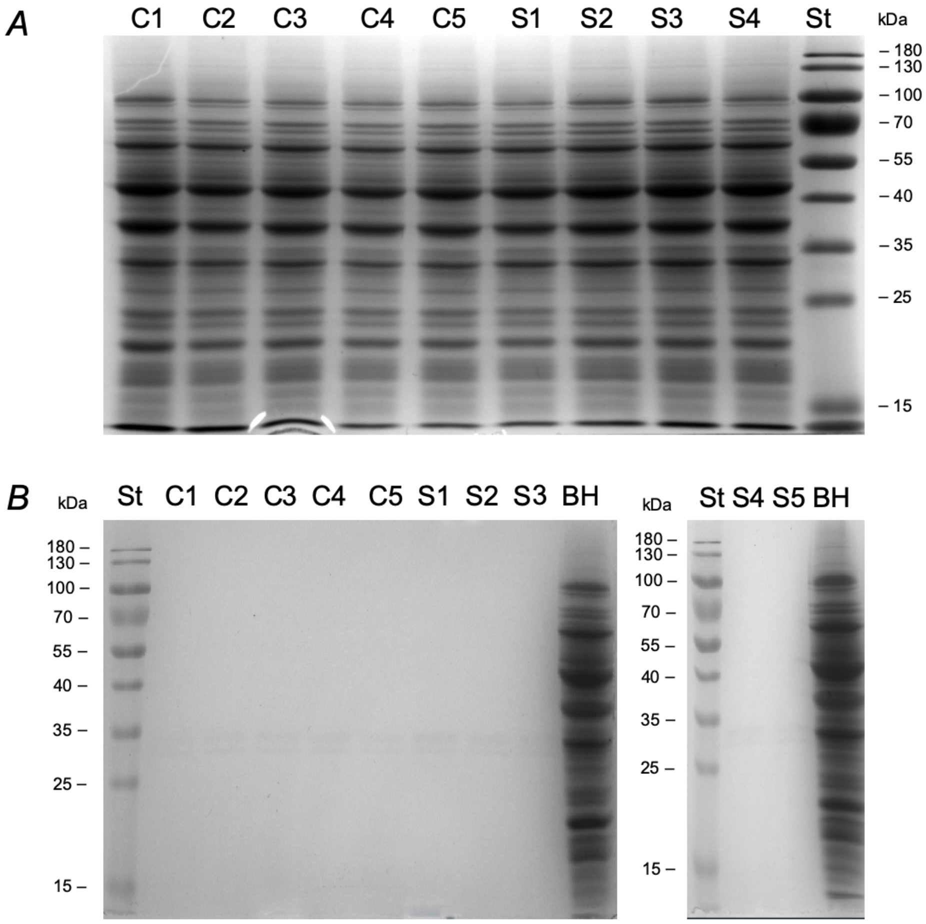

Figure S1-14 SDS-PAGE electropherograms of pea seed protein before (A) and after (B) exhaustive enzymatic hydrolysis (n=5). The aliquots of samples before hydrolysis (10 µg) and aliquots of enzymatic hydrolysates (corresponding to 30 µg of protein) were loaded on the gel in 10 µL of sample buffer. Total protein fraction was isolated from mature seeds of control (C1-C5) and drought-treated (S1 – S5) pea plants; BH, a reference protein sample, not subjected to hydrolysis; St, molecular weight standard mix - Protein Ladder (PageRuler™ Prestained Protein Ladder #26616 (10–180 kDa).

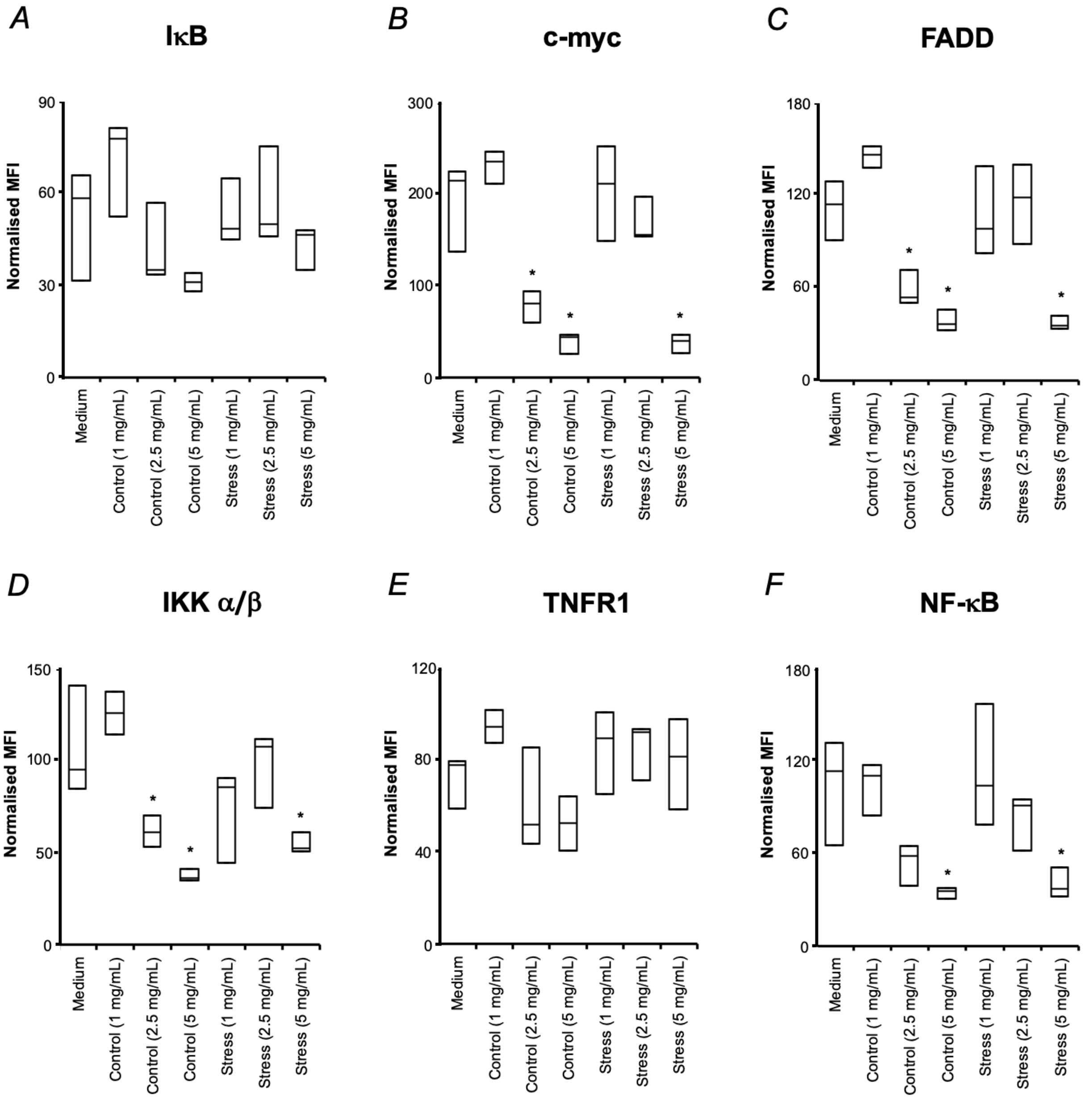

Figure S1-15 The effects of pea seed protein hydrolyzates on the NF-κB-mediated signaling pathway in SH-SY5Y human neuroblastoma cells: optimization of the applied protein amounts. The total protein fraction was isolated from pea (*Pisum sativum* L., cultivar SGE) seeds after a two-day exposure of mature plants (at the seed maturation step) to the aqueous medium with and without addition of 2.5% (w/v) PEG 8000 (defined as Stress and Control, respectively) and subjected to exhaustive enzymatic hydrolysis. The levels of phosphorylated IκB (A), c-myc (B), FADD (C), IKK α/β (D), TNFR1 (E), NF-κB (F) were determined in SH-SY5Y cell lyzates by Luminex® xMAP® technology after 0.5 h incubations with protein hydrolysates (1, 2.5 and 5 mg/mL), supplemented to the culture medium, and normalized to total protein content. The data

are presented as median, inter-quartile range, minimal and maximal values, and were analyzed by one-way ANOVA with Tukey's multiple comparisons test (n=3). The differences in comparison to the medium-treated cells (control), statistically significant at the confidence level $p < 0.05$, are denoted as *.

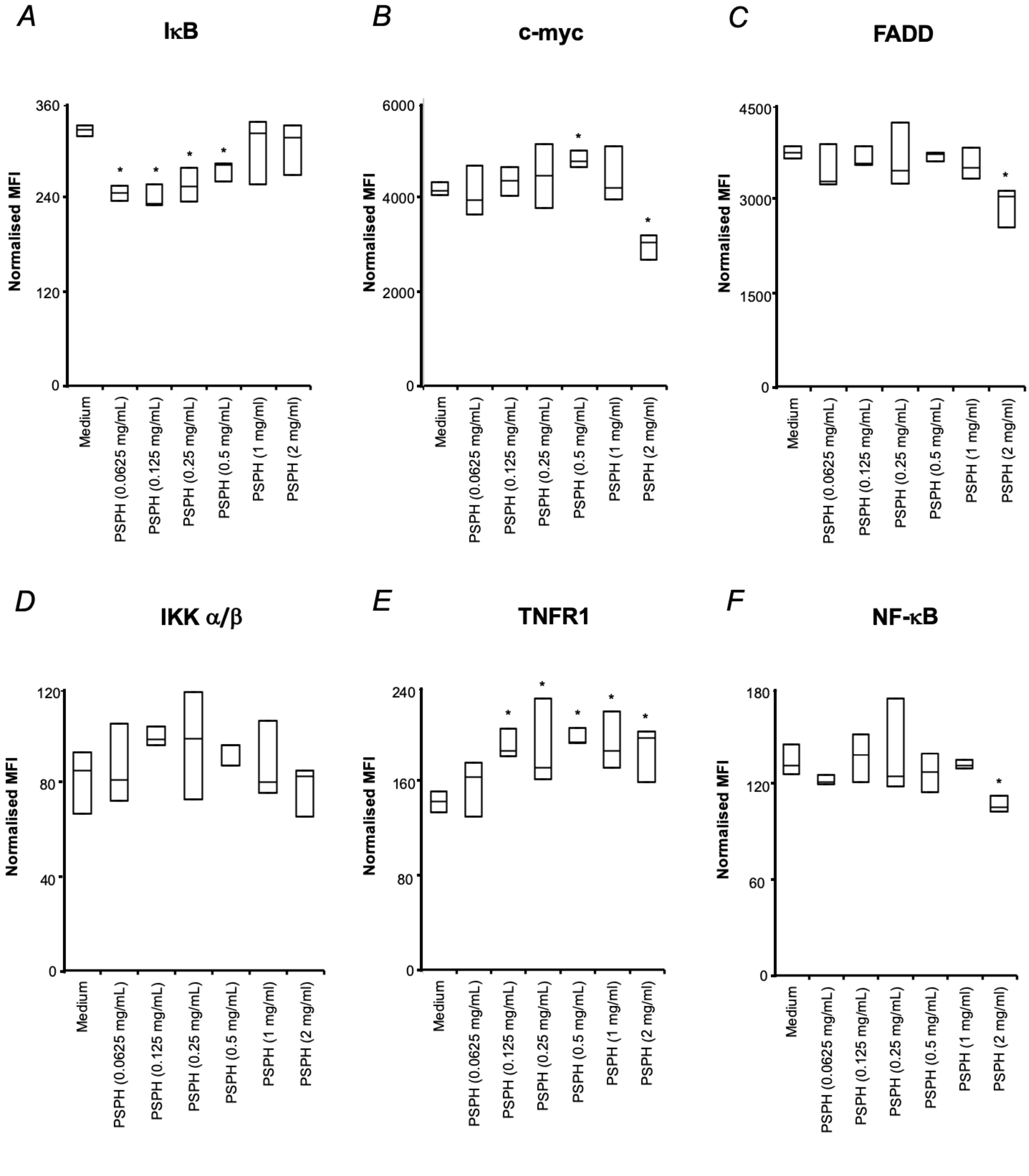

Figure S1-16 The effects of pea seed protein hydrolyzates on the NF-κB-mediated signaling pathway in SH-SY5Y human neuroblastoma cells: optimization of the applied protein amounts. The total protein fraction was isolated from the seeds obtained from mature pea (*Pisum sativum* L.) plants and subjected to exhaustive enzymatic hydrolysis. The levels of phosphorylated IκB (A), c-myc (B), FADD (C), IKK α/β (D), TNFR1 (E), NF-κB (F) were determined in SH-SY5Y cell lyzates by Luminex® xMAP® technology after 0.5 h incubations with protein hydrolysates (0.0625, 0.125, 0.25, 0.5, 1.0 and 2 mg/mL), supplemented to the culture medium, and

normalized to total protein content. The data are presented as median, inter-quartile range, minimal and maximal values, and were analyzed by one-way ANOVA with Tukey's multiple comparisons test (n=3). The differences in comparison to the medium-treated cells (control), statistically significant at the confidence level $p<0.05$, are denoted as *.

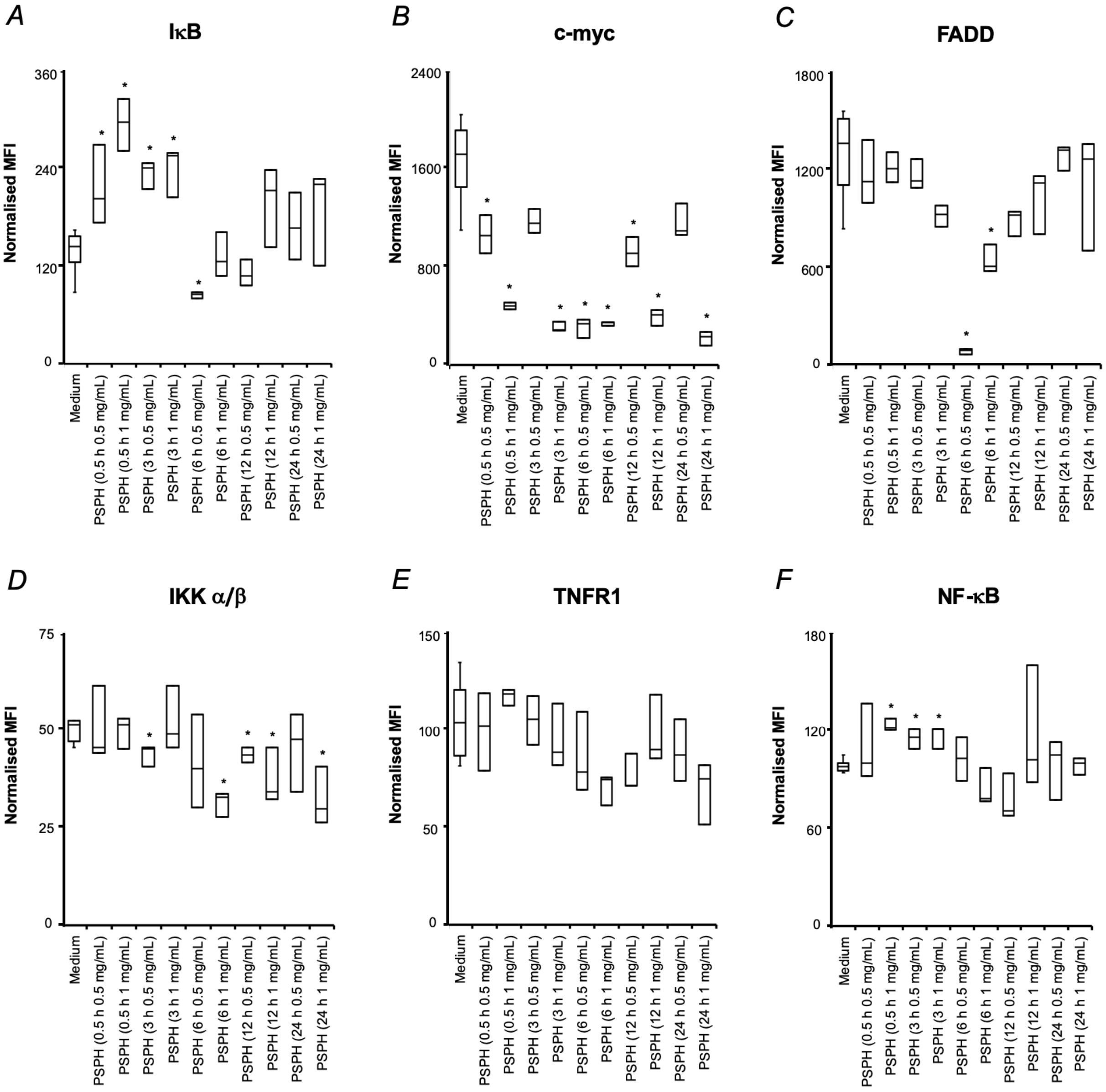

Figure S1-17 The effects of pea seed protein hydrolyzates on the NF-κB-mediated signaling pathway in SH-SY5Y human neuroblastoma cells: optimization of incubation times. The total protein fraction was isolated from the seeds obtained from mature pea (*Pisum sativum* L.) plants and subjected to exhaustive enzymatic hydrolysis. The levels of phosphorylated IκB (A), c-myc (B), FADD (C), IKK α/β (D), TNFR1 (E), NF-κB (F) were determined in SH-SY5Y cell lyzates by Luminex® xMAP® technology after 0.5, 3, 6, 12 and 24 h of incubation with protein hydrolysates (0.5 and 1.0 mg/mL), supplemented to the culture medium, and normalized to total protein content. The data are presented as median, inter-quartile range, minimal and maximal values, and were analyzed by one-way ANOVA with Tukey's multiple comparisons test (n=3).

The differences in comparison to the medium-treated cells (control), statistically significant at the confidence level $p<0.05$, are denoted as *.

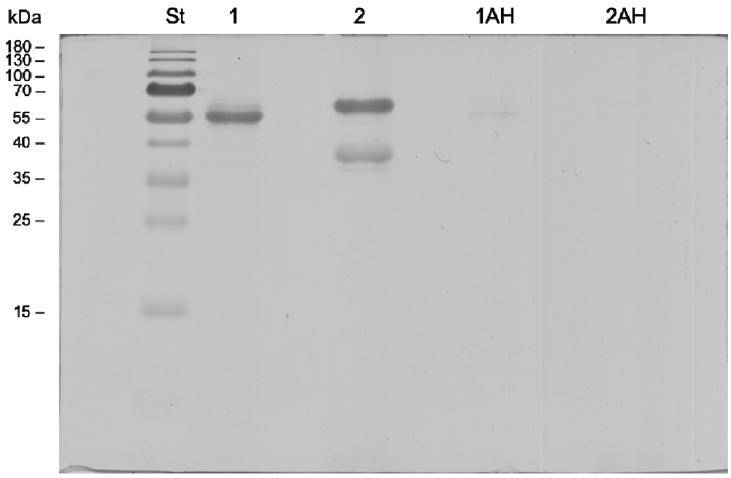

Figure S1-18 SDS-PAGE electropherograms of yeast carboxypeptidase Y purchased from Merck (Sigma-Aldrich GmbH, 1), and carboxypeptidase Y isolated from the yeast *Saccharomyces cerevisiae* according to the protocol of Johansen *et al* (2). The commercial (1AH) and in-house isolated (2AH) preparations could be hydrolyzed with trypsin (> 95% efficiency based on band density); St, Protein Ladder (PageRuler™ Prestained Protein Ladder #26616 (10–180 kDa).

References:

1. Johansen, J.T.; Breddam, K.; Ottesen, M. Isolation of carboxypeptidase Y by affinity chromatography. *Carlsberg Res. Commun.* **1976**, *41*, 1–14.

2. Majovsky, P.; Naumann, C.; Lee, C.-W.; Lassowskat, I.; Trujillo, M.; Dissmeyer, N.; Hoehenwarter, W. Targeted Proteomics Analysis of Protein Degradation in Plant Signaling on an LTQ-Orbitrap Mass Spectrometer. *J. Proteome Res.* **2014**, *13*, 4246–4258.

3. Mamontova, T.; Lukasheva, E.; Mavropolo-Stolyarenko, G.; Proksch, C.; Bilova, T.; Kim, A.; Babakov, V.; Grishina, T.; Hoehenwarter, W.; Medvedev, S.; et al. Proteome Map of Pea (Pisum sativum L .) Embryos Containing Different Amounts of Residual Chlorophylls. *Int. J. Mol. Sci.* **2018**, *19*, 1–20.

4. Mamontova, T.; Afonin, A.M.; Ihling, C.; Soboleva, A.; Lukasheva, E.; Sulima, A.S.; Shtark, O.Y.; Akhtemova, G.A.; Povydysh, M.N.; Sinz, A.; et al. Profiling of seed proteome in pea (pisum sativum l.) lines characterized with high and low responsivity to combined inoculation with nodule bacteria and arbuscular mycorrhizal fungi. *Molecules* **2019**, *24*.

5. Gay, C.; Collins, J.; Gebicki, J.M. Hydroperoxide assay with the ferric-xylenol orange complex. *Anal. Biochem.* **1999**, *273*, 149–155.

6. Greifenhagen, U.; Frolov, A.; Blüher, M.; Hoffmann, R. Site-specific analysis of advanced glycation end products in plasma proteins of type 2 diabetes mellitus patients. *Anal. Bioanal. Chem.* **2016**, *408*, 5557–5566.

7. Antonova, K.; Vikhnina, M.; Soboleva, A.; Mehmood, T.; Heymich, M.-L.; Leonova, T.; Bankin, M.; Lukasheva, E.; Gensberger-Reigl, S.; Medvedev, S.; et al. Analysis of Chemically Labile Glycation Adducts in Seed Proteins: Case Study of Methylglyoxal-Derived Hydroimidazolone 1 (MG-H1). *Int. J. Mol. Sci.* **2019**, *20*, 3659.
